# Mono-Alkylated Ligands Based on Pyrazole and Triazole Derivatives Tested Against *Fusarium oxysporum f*. sp. *albedinis*: Synthesis, Characterization, DFT, and Phytase Binding Site Identification Using Blind Docking/Virtual Screening for Potent Fophy Inhibitors

**DOI:** 10.3389/fchem.2020.559262

**Published:** 2020-12-11

**Authors:** Yassine Kaddouri, Farid Abrigach, Sabir Ouahhoud, Redouane Benabbes, Mohamed El Kodadi, Ali Alsalme, Nabil Al-Zaqri, Ismail Warad, Rachid Touzani

**Affiliations:** ^1^Laboratory of Applied Chemistry and Environment (LCAE), Faculty of Sciences, University Mohammed Premier, Oujda, Morocco; ^2^Laboratory of Biochemistry (LB), Department of Biology, Faculty of Sciences, University Mohamed Premier, Oujda, Morocco; ^3^Centre Régional des Métiers de l'Education et de Formation Oujda, Oriental, Morocco; ^4^Department of Chemistry, College of Science, King Saud University, Riyadh, Saudi Arabia; ^5^Department of Chemistry, College of Science, Ibb University, Ibb, Yemen; ^6^Department of Chemistry, Science College, An-Najah National University, Nablus, Palestine

**Keywords:** pyrazole, triazole, *Fusarium oxysporum*, antifungal, DFT, docking

## Abstract

Twelve recent compounds, incorporating several heterocyclic moieties such as pyrazole, thiazole, triazole, and benzotriazole, made in excellent yield up to 37–99.6%. They were tested against *Fusarium oxysporum f*. sp. *albedinis* fungi (Bayoud disease), where the best results are for compounds **2**, **4**, and **5** with IC_50_ = 18.8–54.4 μg/mL. Density functional theory (DFT) study presented their molecular reactivity, while the docking simulations to describe the synergies between the trained compounds of dataset containing all the tested compounds (57 molecules) and *F. oxysporum* phytase domain (Fophy) enzyme as biological target. By comparing the results of the docking studies for the Fophy protein, it is found that compound **5** has the best affinity followed by compounds **2** and **4**, so there is good agreement with the experimental results where their IC_50_ values are in the following order: 74.28 (**5**) < 150 (**2**) < 214.10 (**4**), using Blind docking/virtual screening of the homology modeled protein and two different tools as Autodock Vina and Dockthor web tool that gave us predicted sites for further antifungal drug design.

## Introduction

*Fusarium oxysporum f*. sp. *albedinis* (F.o.a.) displays the leading dangerous agent among all pathogens of date palm plant, notably in North Africa (Freeman and Maymon, 2000). Thus, infections appear in the vascular wilt of *Phoenix dactylifera*. It is also called Bayoud disease, which is frequently fatal and kills plants in 6 months to 2 years. In the horticultural field, *F. oxysporum* is one of the important fungus organisms raised in cultivated lands. It makes up 40–70% of the entire fusarial flora. It is represented by several diversified forms in terms of morphology and physiology. These forms are saprophytes or parasites of many plants and represent various levels of virulence. Bayoud disease has destroyed more than 15 million Moroccan and Algerian *P. dactylifera* trees (Diana et al., 1995; Hakkou et al., 2004). Unfortunately, to this time, no remedial treatment exists against this fungus, except some limited methods such as land disinfection (Thangavelu and Gopi, 2015), resistant strains propagation (Joshi, 2018), and practice, which have a significance because they reduce this disease's impact. One of the important five-membered hetero atomic rings, where nitrogen and sulfur are separated by one carbon, is 1,3-thiazole, prepared by original strategies. It is a pharmacophore, a privileged scaffold, in many compounds with several biological activities (Reis et al., 2011; Fadda et al., 2012; Alegaon et al., 2014; Bekhit et al., 2015; Varghese et al., 2016; El-Naggar and Abdel-Mottaleb, 2017; El-Sayed and Ismail, 2019; Nayak and Gaonkar, 2019; Pricopie et al., 2019). Pyrazole is a five-membered heterocyclic with two adjacent nitrogen atoms, common in a variety of commercial compounds applied in many industrial fields (Pongor et al., 2004; Elayyachy et al., 2005; Bouabdallah et al., 2006; Dawood and Abdel-Wahab, 2012; Abrigach et al., 2014; Singh et al., 2014, 2016; Akhtar et al., 2017). Another five-membered heterocyclic is 1,2,4-triazole; has three nitrogen atoms at positions 1, 2, and 4 of the ring; and used as a pharmacophore core linked to other compounds, offering different pharmacological activities (Touzani et al., 2003; Sahu et al., 2013; Barbuceanu et al., 2014; Cetin and Gecibesler, 2015; Elbelghiti et al., 2016; Shaikh et al., 2016; Singh et al., 2016; Dalloul et al., 2017). The pyridine may be a six-membered heterocyclic containing merely one nitrogen atom and again documented for several applications (Fadda et al., 2012; Hu et al., 2014; Sun et al., 2014; El-Naggar and Abdel-Mottaleb, 2017; Wei et al., 2019). The pyrimidine is a six-membered heterocyclic having two nitrogen atoms at positions 1 and 3 of the ring, and it has been of significant interest in many applications (Gatta et al., 1990; Iaroshenko et al., 2011; El-Adasy, 2017; Thangarasu et al., 2019). However, because of insufficient information on the pathogenesis of the F.o.a. fungus, numerous computational approaches have usually used to understand further the mechanism of action for this disease antifungal. In this background, docking simulation (Prabhudeva et al., 2019) remains one of the most powerful tools to give an atomistic insight into molecular recognition by predicting the strength of molecule protein–binding modes. The chosen target is the *F. oxysporum* phytase domain (Fophy) enzyme, a protein that plays versatile roles in agricultural and feeding fields. It catalyzes the degradation of phytate (an essential constituent of grains, cereals, and oilseeds) into inorganic phosphorus and myoinositol phosphate derivatives. Inhibition of the Fophy enzyme can affect the expansion of the fungus indirectly by preventing the phytate degradation, well-established as a robust chelating agent readily binding to covalent metal ions and renders them insoluble and therefore unavailable for absorption. These characteristics made both these proteins prospective potential targets to develop new anti-*F. oxysporum* inhibitors (Gontia-Mishra et al., 2014). And keeping in mind the biological significance of heterocyclic ligands, we described the synthesis of new heterocyclic compounds, used as potent antimicrobial agents, in our study. A molecular docking approach was used for the best antifungal derivatives against Fophy. The structure of this approach was constructed using the homology model that has been previously reported in the literature (Soundararajan et al., 2011; Abrigach et al., 2014; Tighadouini et al., 2019; Toubi et al., 2019), to achieve better insight into the ligand–receptor binding interactions and direct future synthesis. In case that there is no cofactor, blind docking, and virtual screening are used in this study for site prediction and protocol validation using Autodock Vina (Seeliger and de Groot, 2010) and Dockthor (Santos et al., 2020).

## Materials and Methods

### Chemicals and Instruments

All the chemicals used in this study were of analytical grades (Aldrich, purity >99%). Melting points were measured with Koffler bank and the FTIR analysis with the FTIR-8400S spectrometer using KBr pellets. We recorded the ^**1**^H and ^**13**^C nuclear magnetic resonance (NMR) spectra on AVANCE 300, 400, and 500 MHz from BRUKER. The *in vitro* anti-*Fusarium* activity was tested by the agar diffusion technique.

### Synthesis of the Pyrazole and Triazole

#### General Procedure for Preparing Compounds 1–12

**Figure 1:**
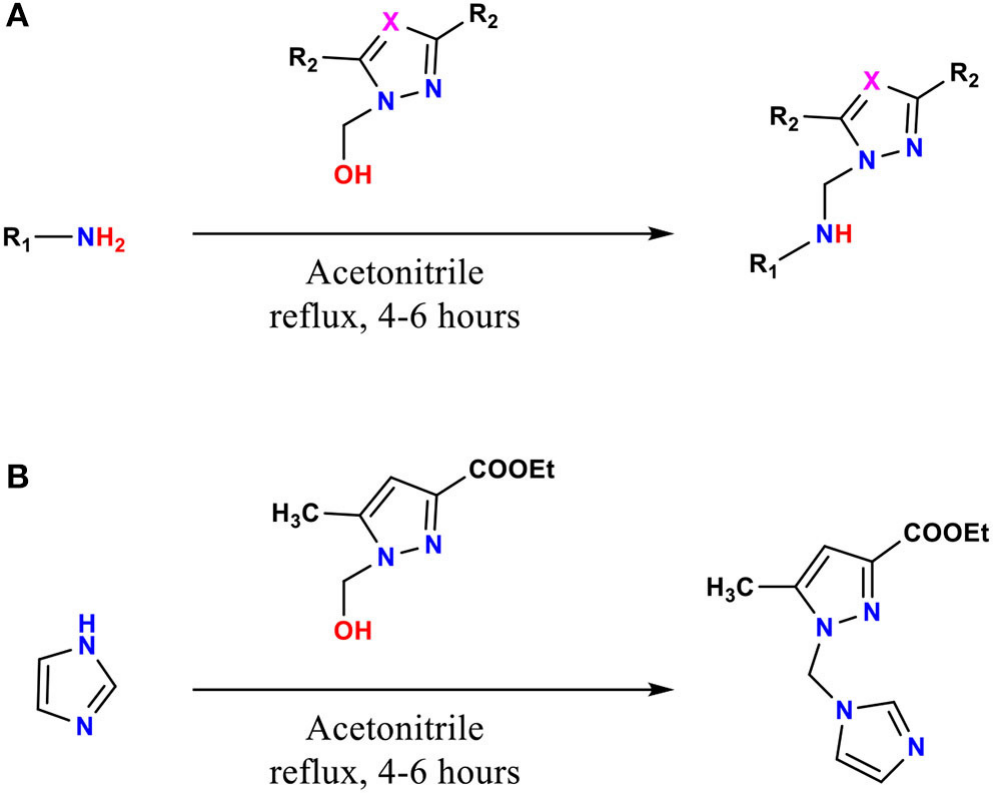
The reaction procedure for the preparation of compounds **1**–**12** (pathway **A,B**).

The ligands **1**–**9**, **11**, and **12** were prepared by condensation of different monoamines with pyrazole or 1,2,4-triazole methanol derivatives (Pathway A, Figure 1), whereas **10** were prepared by condensation of imidazole with ethyl 1-(hydroxymethyl)-5-methyl-1H-pyrazole-3-carboxylate (Pathway B, Figure 1), according to the method described in the literature (Kaddouri et al., 2017, 2019, 2020).

**2-(((1H-1,2,4-triazol-1-yl)methyl)amino) nicotinic acid, 1:** Viscous product; ^1^H NMR [500 MHz, dimethyl sulfoxide (DMSO)-d_6_]: δ 8.33 (m, 1H), 7.09 (s, 4H), 4.94 (s, 2H); ^13^C NMR (125 MHz, DMSO-d_6_): δ 168.54, 159.67, 151.10, 144.17, 140.26, 113.46, 111.81, 54.

**N-((3,5-dimethyl-1H-pyrazol-1-yl) methyl)pyridin-2-amine, 2:** Yield, 89.85%; mp 122–124°C; ^1^H NMR (500 MHz, CDCl_3_): δ 8.03 (d, *J* = 5.1 Hz, 1H), 7.33–7.29 (m, 1H), 6.56 (d, J = 7.2 Hz, 1H), 6.46 (d, *J* = 8.3 Hz, 1H), 5.51 (s, 1H), 5.48 (s, 1H), 2.34 (s, 3H), 2.13 (s, 1H), 2.10 (s, 3H); ^13^C NMR (125 MHz, CDCl_3_): δ 156.53, 148.18, 147.39, 139.70, 137.59, 114.31, 109.06, 105.29, 54.42, 13.49, 11.12; CG-MS: m/z (%) = 281.2 [M+DMSO] ^+^ [calcd. for C_11_H_14_N_4_ [M+DMSO] ^+^ 280.26].

**N-((3,5-dimethyl-1H-pyrazol-1-yl) methyl)-6-methylpyridin-2-amine, 3:** Yield, 30%; mp: 136°C−138°C; ^1^H NMR (500 MHz, CD_2_Cl_2_): δ 7.42 (t, *J* = 10 Hz, 1H), 7.21 (dd, *J* = 10 Hz, 1H), 6.40 (d, *J* = 5 Hz, 1H), 6.25 (d, *J* = 10 Hz, 1H), 5.64 (s, 1H), 5.43 (s, 2H), 2.36 (s, 3H), 2.27 (s, 3H), 2.06 (s, 3H), ^13^C NMR (125 MHz, CD_2_Cl_2_) δ: 157.01, 155.95, 146.57, 139.51, 137.98, 112.49, 105.95, 104.97, 53.79, 24.39, 13.78, 11.17; CG-MS: m/z (%) = 281.2 [M+ACN+Na]^+^ [calcd. for C_12_H_16_N_4_ [M+ACN+Na]^+^ 280.29].

**N-((1H-1,2,4-triazol-1-yl)methyl)-5-bromopyridin-2-amine, 4:** Yield, 62.14%; mp: 64–66°C; ^1^H NMR (500 MHz, DMSO-d_6_): δ 8.56 (s, 1H), 8.15 (s, 1H), 7.95 (s, 1H), 6.64 (d, *J* = 8.9 Hz, 1H), 5.67 (d, *J* = 6.9, 1H); ^13^C NMR (125 MHz, DMSO-d_6_) δ: 155.68, 151.11, 147.63, 144.07, 139.78, 111.06, 107.64, 54.55; CG-MS: m/z (%) = 370.9 [M+2ACN+CH_3_OH+2H]^+^ [calcd. for C_8_H_8_BrN_5_ [M+2ACN+CH_3_OH+2H]^+^ 370.09].

**N-((1H-pyrazol-1-yl)methyl)-5-bromopyridin-2-amine, 5** (Abrigach et al., 2014): Yield, 62.36%; mp: 114–116°C; ^1^H NMR (500 MHz, DMSO-d_6_): δ 8.14 (s, 1H), 7.98 (t, *J* = 6.9 Hz, 1H), 7.78 (d, *J* = 2.3 Hz, 1H), 7.63 (d, *J* = 8.9 Hz, 1H), 7.44 (d, *J* = 1.8 Hz, 1H), 6.62 (d, *J* = 8.9 Hz, 1H), 6.22 (dd, *J* = 2.3 Hz, 1H), 5.60 (d, *J* = 6.9 Hz, 2H); ^13^C NMR (125 MHz, DMSO-d_6_): δ 156.11, 147.63, 139.62, 138.55, 129.47, 110.78, 107.16, 105.16, 56.44; CG-MS: m/z (%) = 372.8 [M+DMSO+K+2H]^+^ [calcd. for C_9_H_9_BrN_4_ [M+DMSO+K+2H]^+^ 372.1].

**5-Bromo-N-((3,5-dimethyl-1H-pyrazol-1-yl)methyl) pyridin-2-amine, 6** (Abrigach et al., 2014): Yield, 93.6%; mp: 150–152°C; ^1^H NMR (500 MHz, DMSO-d_6_): δ 8.11 (s, 1H), 7.84 (t, *J* = 6.6 Hz, 1H), 7.60 (d, *J* = 8.9 Hz, 1H), 6.64 (d, *J* = 8.9 Hz, 1H), 5.76 (s, 1H), 5.42 (d, J = 6.6 Hz, 2H), 2.56 (s, 3H), 2.34 (s, 3H); ^13^C NMR (125 MHz, DMSO-d_6_): δ 156.06, 147.44, 139.47, 110.66, 101.75, 53.42, 13.29, 10.67; CG-MS: m/z (%) = 281.0 [M]^+^ [calcd. for C_11_H_13_BrN_4_ [M]^+^ 281.16].

**N-((1H-pyrazol-1-yl)methyl)thiazol-2-amine, (****Figure 3****):** Yield, 53.08%; mp: 108–110°C; ^1^H NMR (500 MHz, DMSO-d_6_) in Figure 4: δ 8.68 (t, *J* = 6.7 Hz, 1H), 7.80 (d, *J* = 2.3 Hz, 1H), 7.46 (d, *J* = 1.1 Hz, 1H), 7.09 (d, *J* = 3.6 Hz, 1H), 6.74 (d, *J* = 3.6 Hz, 1H), 6.23 (t, *J* = 2.0 Hz, 1H), 5.56 (s, 2H); ^13^C NMR (125 MHz, DMSO-d_6_) in Figure 5: δ 168.11, 139.33, 139.02, 130.47, 108.52, 105.76, 59.40, CG-MS: m/z (%) = 213 [M+CH_3_OH]^+^ [calcd. for C_7_H_8_N_4_S [M+CH_3_OH]^+^ 212.23].

**N-((3,5-dimethyl-1H-pyrazol-1-yl)methyl)thiazol-2-amine, 8:** Yield, 37.12%; mp: 152–154°C; ^1^H NMR (500 MHz, CDCl_3_): δ 7.05 (d, *J* = 3.6 Hz, 1H), 6.78 (s, 2H), 6.44 (d, *J* = 3.6 Hz, 1H), 5.70 (s, 1H), 5.45 (s, 2H), 2.35 (s, 3H); 2.14 (s, 3H); ^13^C NMR (125 MHz, CDCl_3_): δ 167.52, 148.68, 140.07, 138.67, 108.07, 105.57, 56.74, 13.46, 11.11; CG-MS: m/z (%) = 293.1 [M+2ACN+2H]2 ^+^ [calcd. for C_9_H_12_N_4_S [M+2ACN+2H]^+^ 292.28].

**N-((3,5-dimethyl-1H-pyrazol-1-yl)methyl)pyridin-4-amine, 9** (Abrigach et al., 2014): Yield, 29.79%; mp: 154–156°C; ^1^H NMR (300 MHz, MeOD): δ 8.06 (d, J = 1.5 Hz, 2H), 6.81 (d, *J* = 1.5 Hz, 2H), 6.79 (t, *J* = 1.5 Hz, 1H), 6.55 (s, 1H), 5.39 (s, 2H), 2.32 (s, 3H), 2.17 (s, 3H); ^13^C NMR (75 MHz, MeOD): δ 155.50, 148.49, 147.45, 139.91, 108.89, 105.87, 65.58, 11.91, 9.64; CG-MS: m/z (%) = 281.4 [M+DMSO+H]^+^ [calcd. for C_11_H_14_N_4_ [M+DMSO+H]^+^ 281.26].

**Ethyl 1-((1H-imidazol-1-yl)methyl)-5-methyl-1H-pyrazole-3-carboxylate, 10:** Yield, 99.6%; mp: 74–76°C; ^1^H NMR (500 MHz, DMSO-d_6_): δ 7.68 (d, *J* = 1.0 Hz, 1H), 7.04 (d, *J* = 1.0 Hz, 3H), 6.50 (d, *J* = 0.9 Hz, 2H), 4.26 (q, *J* = 7.1 Hz, 2H), 2.26 (s, 3H), 1.28 (s, 3H); ^13^C NMR (125 MHz, DMSO-d_6_): δ 161.63, 135.11, 121.64, 106.56, 59.89, 14.14, 10.68; CG-MS: m/z (%) = 310 [M+ACN+CH_3_OH+H]^+^ [calcd. for C_11_H_14_N_4_O_2_ [M+ACN+CH_3_OH+H]^+^ 309.26].

**2-(((1H-pyrazol-1-yl)methyl)amino)-6-methylpyridin-4-ol, 11** (Abrigach et al., 2014): Yield, 93.82%; mp: 238–240°C, ^1^H NMR (500 MHz, DMSO-d_6_): δ 11.3 (s, 1H), 6.71 (s, 1H), 6.11 (s, 2H), 5.18 (s, 2H), 1.89 (s, 3H), 1.80 (m, 1H), 1.76 (s, 3H), 1.63 (s, 3H); ^13^C NMR (125 MHz, DMSO-d_6_): δ 164.80, 155.47, 153.96, 138.96, 129.30, 105.64, 100.34, 73.24, 23.60; CG-MS: m/z (%) = 234.1 [M+H]^+^ [calcd. for C_11_H_15_N_5_O [M+H]^+^ 233.28].

**2-(((3,5-dimethyl-1H-pyrazol-1-yl)methyl)amino)-6-methylpyridin-4-ol, 12:** Yield, 90.65%; mp: 100–102°C, ^1^H NMR (500 MHz, DMSO-d_6_): δ 11.00 (s, 1H), 7.76 (t, *J* = 2.3 Hz, 1H), 7.46 (t, *J* = 2.1 Hz, 1H), 5.56 (d, *H* = 12 Hz, 1H), 5.39 (s, 1H), 5.36 (d, *J* = 3.5 Hz, 1H), 1.98 (s, 3H); ^13^C NMR (125 MHz, DMSO-d_6_): δ 175.56, 172.05, 171.78, 163.62, 161.41, 99.54, 25.16, 23.20, 22.41; CG-MS: m/z (%) = 206.1 [M+H]^+^ [calcd. for C_9_H_11_N_5_O [M+H]^+^ 205.22].

### Biological Evaluation

#### Anti-*Fusarium* Assay

First, the F.o.a. was isolated from Bouffagous Gharas date palm from Figuig in Morocco that is infected by the vascular fusariosis according to the protocol described by Benabbes et al. (2015), who followed the protocol of Locke and Colhoun (1974). Then, DMSO solution of each ligand was made at a concentration (4 mg/mL) and was thus employed for the preparation of various concentrations from potato dextrose agar (PDA) solutions with different volumes (50, 160, and 500 μL). The Petri plates were prepared with 8.7-cm diameter using 10 mL (Neri et al., 2006). After that, cultivated F.o.a. was transplanted onto the solid PDA with a pellet form in each plate center and again was incubated at 28°C for 5 days. The results were expressed in percentage (%) of inhibition, calculated from the measured width of F.o.a., compared to the positive control having only DMSO, which has no inhibition on F.o.a. (Hmouni et al., 1996). The experiments are repeated three times (three independent experiments, *n* = 3 with SEM ±).

$$\begin{array}{l} \text{\% of inhibition}=\frac{\left(D_{o}-D_{x}\right)}{D_{o}}\times 100 \end{array}$$

where *D*_0_ = diameter in centimeters of F.o.a. in the control and *D*_x_ = diameter in centimeters of F.o.a. in the test.

Positive control: PDA+500 μL of DMSO; negative control: PDA+F.o.a.

After that, several tests were done to find the volume of the ligand to 50% of inhibition experimentally not doing the linear regression, with the objective to calculate the concentration of ligand needed to inhibit 50% of the F.o.a. (Radi et al., 2015; Tighadouni et al., 2016; Koudad et al., 2019; Tighadouini et al., 2020).

### Theoretical Investigations

#### DFT Calculations

The DFT study was performed using Gaussian 09W software (Frisch et al., 2009) by the DFT (Eschrig, 2003; Capelle, 2006; Van Mourik et al., 2014; Domingo et al., 2016; Contreras-García and Yang, 2018) method with three functional parameters of Becke associated to the functional correlation gradient corrected by Lee Yang Parr (B3LYP) (Becke and Becke, 1993; Becke, 2014) and the exchange correlation in combination with 6–31 G (d, p) orbital basis sets for all atoms, with no symmetrical constrains. The molecular electrostatic potential (MEP) surfaces are generated in default parameters as total density and electrostatic potential (ESP) with the self-consistent field matrix for cubes and surface map generation.

#### Ligand Preparation

The compounds of interest for further docking purposes are geometrically optimized applying the DFT method, and the frequency was calculated for ground state verification after saving them as mole files later included in open label to convert them into pdbqt files that will be incorporated in Autodock Vina for virtual screening.

#### Protein Preparation and Active Site Selection

The modeled proteins structure of Fophy considered as target reported in the literature (Abrigach et al., 2018; Kaddouri et al., 2019; Tighadouini et al., 2019; Toubi et al., 2019) was prepared in Autodock 4 default parameters, and the whole protein was used as grid for blind docking–virtual screening (Table 1) with Perl as launcher of virtual screening for all the ligands in Autodock Vina (Seeliger and de Groot, 2010).

**Table 1:** Blind docking simulation parameters and virtual screening configuration using Autodock Vina.

Docking parameters	• Genetic algorithm • 2,500,000 no. of evals (medium) • Lamarckian V4 output
Perl configuration	#!/usr/bin/perl print“Ligand_file:\t”; $ligfile = <STDIN>; chomp $ligfile; open (FH,$ligfile)||die “Cannot open file\n”; @arr_file = <FH>; for($i=0;$i < @arr_file;$i++) { print“@arr_file[$i]\n”; @name=split(/\./,@arr_file[$i]); } for($i=0;$i < @arr_file;$i++) { chomp @arr_file[$i]; print“@arr_file[$i]\n”; system(“vina.exe –config conf_vs.txt –ligand @arr_file[$i] –log @arr_file[$i]_log.log”); }
Virtual screening configuration	• **center_x =** −5.932 • **center_y =** −1.368 • **center_z =** 29.033 • **size_x =** 80 • **size_y =** 68 • **size_z =** 56 • **num_modes =** 10 • **energy_range =** 4

For the docking validation, Dockthor (Santos et al., 2020), a web tool for ligand–protein docking, was used for blind docking/virtual screening of the active compounds in the whole Fophy protein chain, and the parameters for this is described in Table 2.

**Table 2:** Blind docking simulation parameters and virtual screening configuration using Dockthor.

Virtual screening configuration	• **center_x =** −3.018 • **center_y =** −1.2215 • **center_z =** 27.7495 • **size_x =** 40 • **size_y =** 40 • **size_z =** 40
Search algorithm	• **No. of evaluations:** 500,000 • **Population size:** 750 • **Initial seed:** −1,985 • **No. of runs:** 12 • **Soft docking**

#### Dataset Preparation

The selected compounds are all the testes against F.o.a., collected from the references (Waring et al., 2002; Radi et al., 2012, 2015; Smaail et al., 2012; Boussalah et al., 2013; Loth et al., 2015; Abrigach et al., 2017, 2018; Tighadouini et al., 2018, 2019; Koudad et al., 2019; Toubi et al., 2019).

In Table 3, this datatset contained 57 compounds, where 6 of them are tested against phytase inhibitors with Ki values that are converted to pKi between 3.16 for Dichlorvos and 4.50 for crytoxyphos. The dataset compounds structure is collected in with their pKi, IC_50_, or MIC values.

**Table 3:**
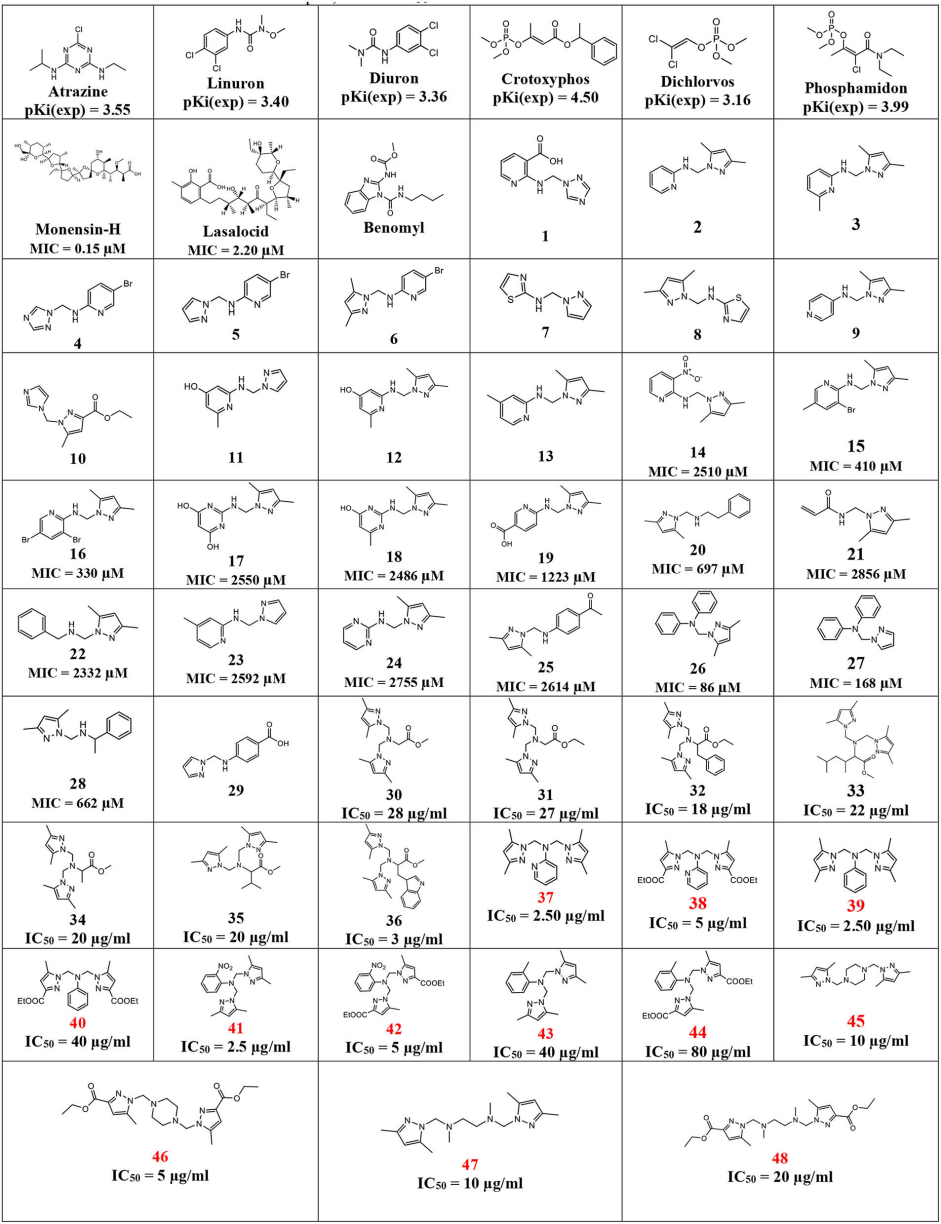
Dataset structures with their pKi, MIC, or IC_50_ values.

*MIC, minimum inhibitory concentration; IC50, half maximal inhibitory concentration, pKi, inhibition constant*.

#### ADME Predictions

The ADME (adsorption, distribution, metabolism, and exertion) properties are depicted from SwissADME web tool (Hou et al., 2004; Arnott and Planey, 2012; Daina et al., 2017).

## Results and Discussion

### Chemistry

**Figure 2:**
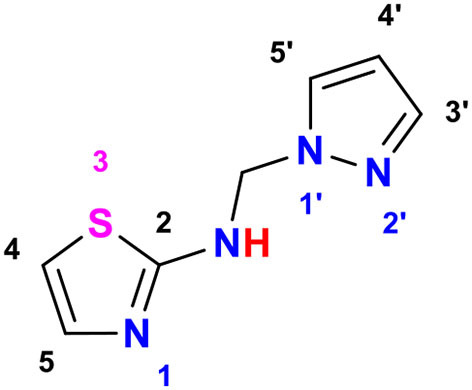
The chemical structure of N-((1H-pyrazol-1-yl)methyl)thiazol-2-amine, 7.

The mono-alkylated ligands based on pyrazole and 1,2,4-triazole are prepared (Figure 2), whereas compounds **5**, **6**, **9**, and **11** were described in the literature (Touzani et al., 2003; Kaddouri et al., 2019, 2020). Several physicochemical analysis methods that are described in section Synthesis of the Pyrazole and Triazole Ligands characterized the prepared ligands.

All compounds were characterized by ^1^H and ^13^C NMR. Table 4 illustrates the chemical shifts of CH_2_ in the examined ligands, where there is no significant difference between the studied ligands, and their structures are unique.

**Table 4:** The chemical shifts of CH_2_ in the studied ligands.

**Compound**	**^**1**^H NMR (δ (CH_**2**_))**	**^**13**^C NMR (δ (CH_**2**_))**
**1**	5.22	54
**2**	5.52	54.42
**3**	5.43	53.79
**4**	5.67	54.55
**5**	5.59	56.44
**6**	5.76	53.42
**7**	5.56	58.93
**8**	5.45	56.74
**9**	5.39	65.58
**10**	4.25	59.88
**11**	5.18	73.24
**12**	5.18	73.70

The chemical shifts of the mono-alkylated ligands **1**–**12** are located in the regions 5.18–5.76 ppm for ^1^H NMR, indicating a doublet peak that takes place because of the coupling of CH_2_ with the nearest free proton NH, and 53.42–73.7 ppm for ^13^C NMR, except for ligand **10**, which carries an original chemical structure. For ligand **10**, the ^1^H NMR chemical shift is 4.25 ppm with a single peak, and the ^13^C NMR chemical shift is 59.88 ppm.

**Figure 3:**
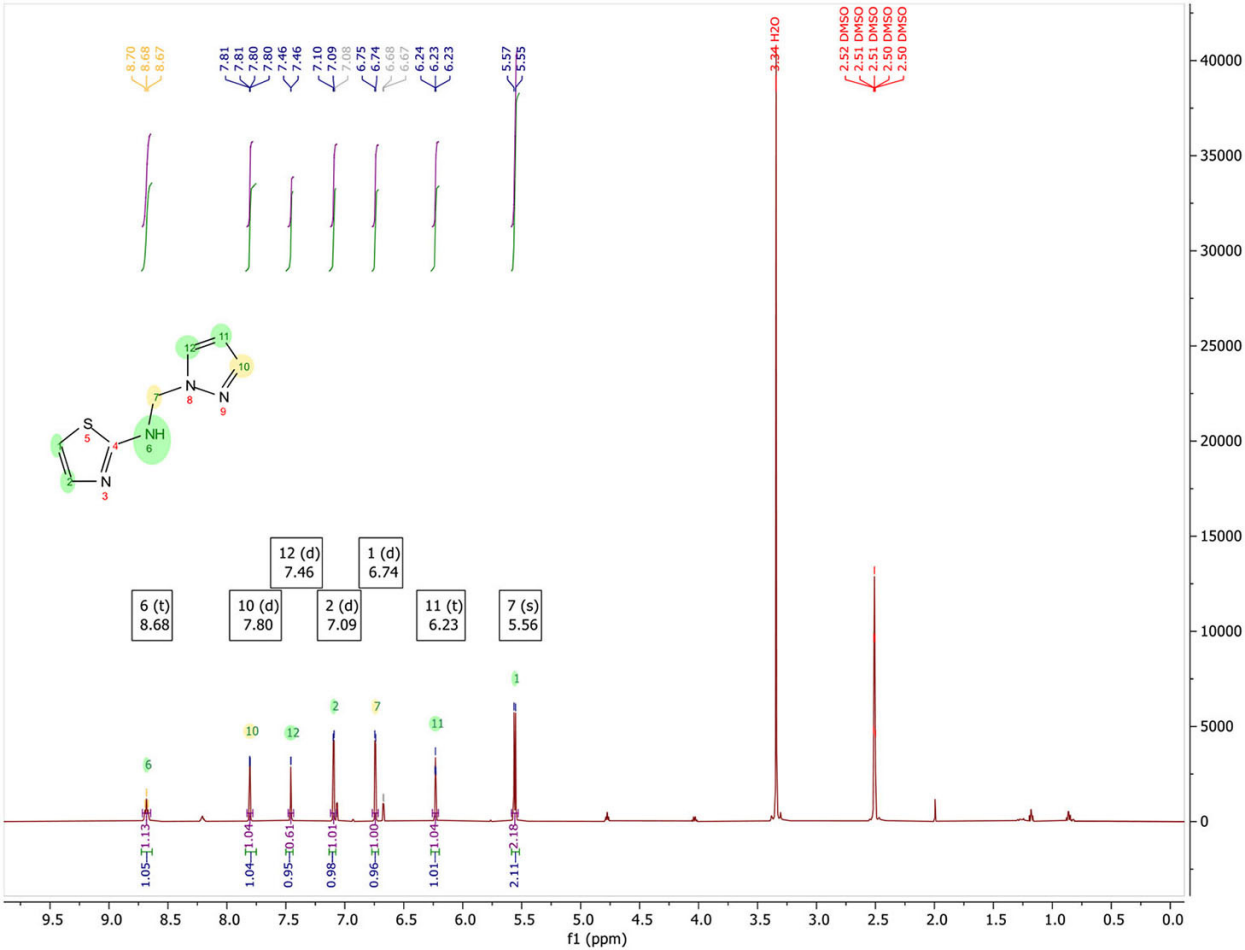
1H NMR spectrum of N-((1H-pyrazol-1-yl)methyl)thiazol-2-amine, 7.

As an example, for the characterization of the studied compounds, N-((1H-pyrazol-1-yl)methyl)thiazol-2-amine, 7 in Figure 3 is displayed as follows:

To formulate this compound, 1.5 g of 2-aminothiazole (14.9 mmol) and 1.47 g of (1H-pyrazol-1-yl) methanol (14.9 mmol) were mixed in acetonitrile under reflux for 4 h, and the solvent was evaporated and then recrystallized in diethyl ether after filtration to have the final product (1.42 g, 53.08%): **mp** 108–110°C.

**Figure 4:**
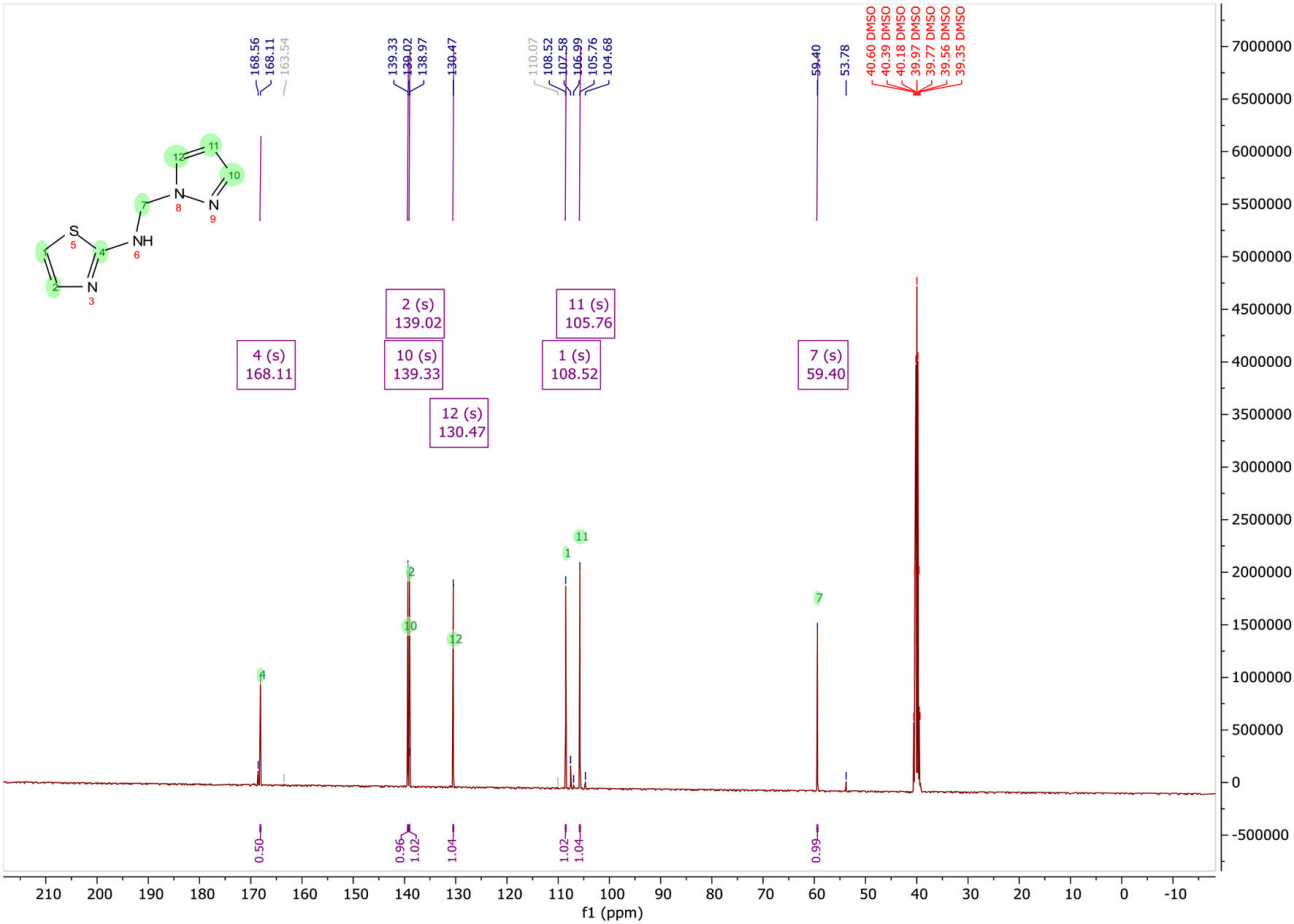
^13^C NMR spectrum of N-((1H-pyrazol-1-yl)methyl)thiazol-2-amine, 7.

In Figure 4, ^1^H NMR (DMSO-d_6_, 500 MHz) spectrum of N-((1H-pyrazol-1-yl)methyl)thiazol-2-amine encloses the following peaks:

- Triplet integration at 8.68 ppm with *J*_H−H_ = 6.7 Hz for the proton of the NH.
- Doublet integration at 7.80 ppm with *J*_H−H_ = 2.3 Hz for the proton in position 3′.
- Doublet integration at 7.46 ppm with *J*_H−H_ = 1.1 Hz for the proton in position 5′.
- Doublet integration at 7.09 ppm with *J*_H−H_ = 3.6 Hz for the proton in position 5.
- Doublet di-doublet integration at 6.74 ppm with *J*_H−H_ = 3.6 Hz for the proton in position 4.
- Triplet integration at 6.23 ppm with *J*_H−H_ = 2.0 Hz for the proton in position 4′.
- Singlet integration at 5.56 ppm for the protons of the CH_2_.

**Figure 5:**
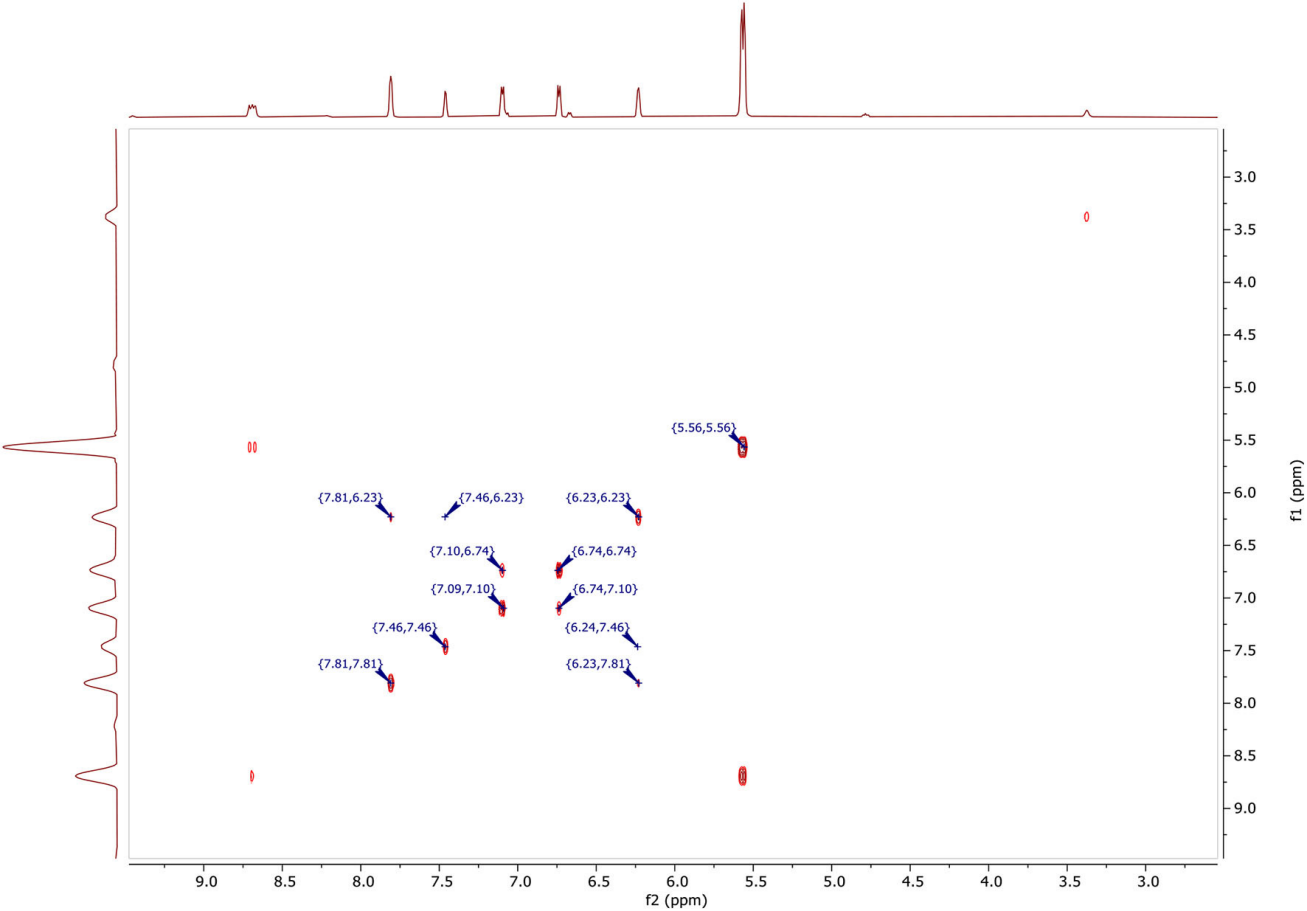
Two-dimensional NMR COSY spectrum of N-((1H-pyrazol-1-yl)methyl)thiazol-2-amine, 7.

In Figure 5, NMR ^13^C (DMSO-d_6_, 125 MHz) of N-((1H-pyrazol-1-yl)methyl)thiazol-2-amine contain the following peaks at:

- 168.11 for the carbone C2;
- 139.33 for the carbone C3′;
- 139.02 for the carbone C5;
- 130.47 for the carbone C5′;
- 108.52 for the carbone C4;
- 105.76 for the carbone C4′;
- 59.40 for the carbone CH_2_.

**Figure 6:**
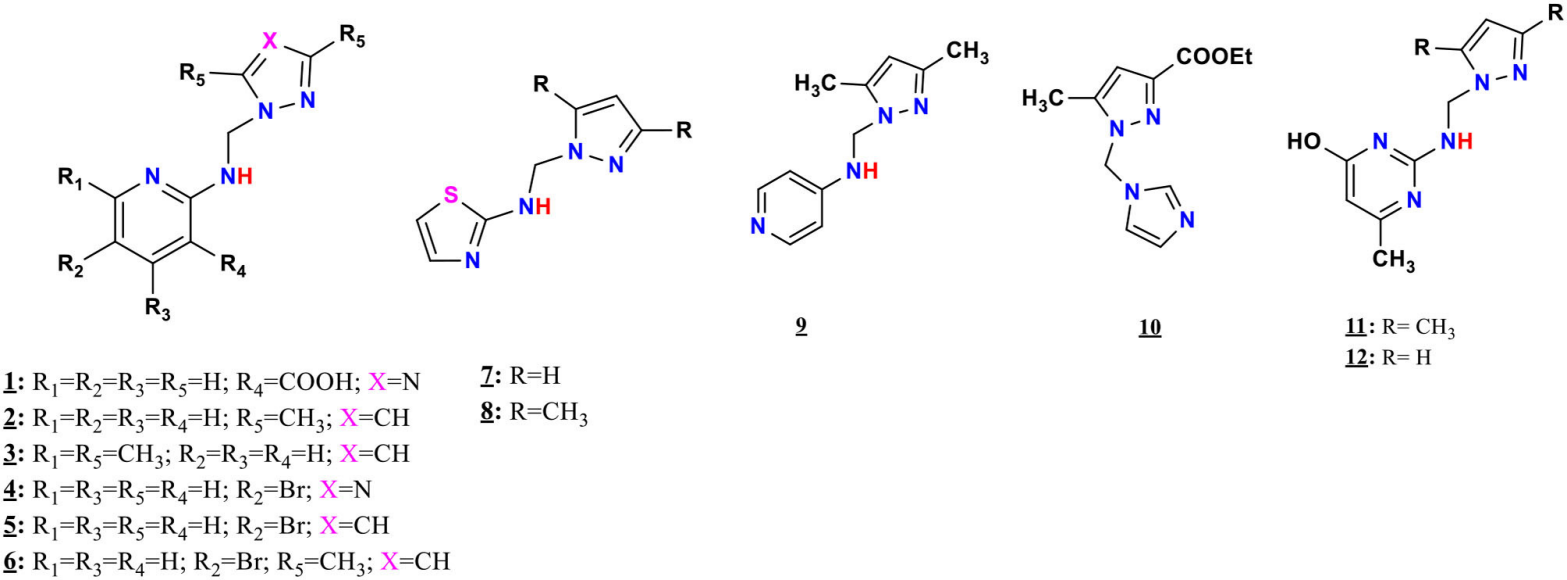
Synthesis route and chemical structures of the mono-alkylated pyrazole and triazole ligands.

In Figure 6, two-dimensional NMR COSY (DMSO-d6, 400 MHz) δ ppm contains the following spots at:

- (5.56, 5.56) for CH_2_.
- (6.23, 6.23), (7.46, 6.23), (7.81, 6.23), (6.24, 7.46), and (6.23, 7.81) for CH (4′).
- (6.71, 6.74), (7.10, 6.74), and (6.71, 7.10) for CH (4).
- (7.09, 7.10), (7.10, 6.74), and (6.71, 7.10) for CH (5).
- (7.46, 7.46), (7.46, 6.23), and (6.24, 7.46) for CH (5′).
- (7.81, 7.81), (7.81, 6.23), and (6.23, 7.81) for CH (3′).

### Biological Assay and Lipophilicity Study

The preliminary results established that ligands **2**, **4**, and **5** displayed a significant inhibitory effect on F.o.a., in volumes over 50 μL, i.e., at concentrations higher than 16 μg/mL. To affirm that effect, we retested those ligands in three independent experiments (*n* = 3 experiments with ±SEM) to get the minimum inhibitory concentrations (MICs) experimentally.

**Figure 7:**
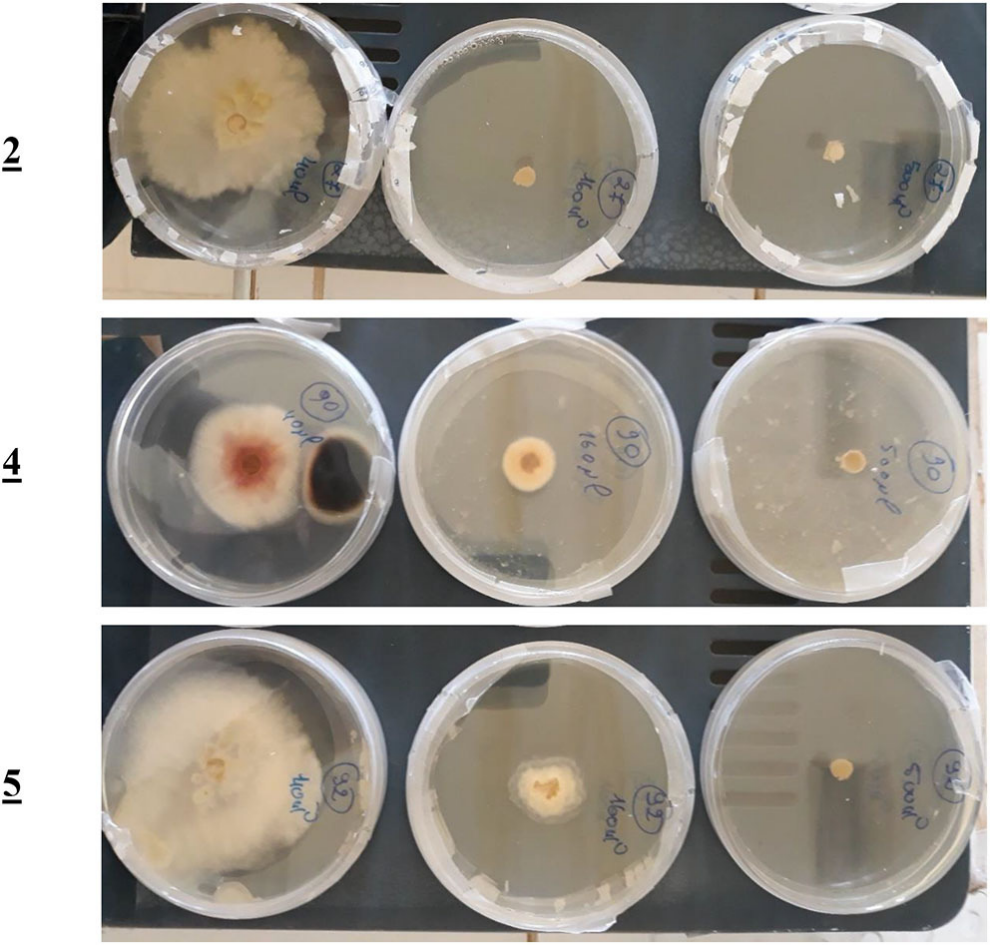
The F.o.a. Petri plates picture for compounds **2**, **4**, and **5**.

For the mono-alkylated ligands, Table 5 demonstrates that ligand **2** carries a 6.7-cm diameter of the strain in 50 μL (Figure 7) that resembled 22.99% inhibition. It kills F.o.a., appearing from 160 μL, at 100% inhibition. Ligand **3** has the same structure as ligand **2** but methyl group at the α position of the pyridine ring. This causes its inactivity, exhibiting no inhibition in 50 μL. Ligand **1** shows no inhibition at all. Ligands **4** (Figure 7), **5**, and **6** show a respectable percentage of inhibition in 50 μL, corresponding to 25.29, 56.32, and 8.05%, respectively. The highest inhibition was seen in ligand **5** with IC_50_ = 18.8 μg/mL (Figure 7); it has no substituents on the pyrazole moiety, contrary to the substituted one; ligand **6** has a less percentage of inhibition. The results also show no meaningful difference, in terms of inhibition efficiency, between ligands **7** and **8**, even if there are two methyl groups at positions 3 and 5 on the pyrazole moiety in compound **8**. Ligands **9** and **10** kill F.o.a. in 500 μL with 100% of inhibition, showing no inhibition in other volumes. Ligands **11** and **12** show acceptable percentages of inhibition, starting from 160 μL, at 28.74%, and 35.63%, respectively, and the finest result obtained is by the non-substituted pyrazole moiety in compound **12**. These compounds have identical or better results than compounds described in the literature by Tighadouini et al. (2019), where their compound **9** has 76% of inhibition in 200 μL, whereas compound **5** has 79.31% in 160 μL.

**Table 5:** The preliminary results of the studied ligands tested against F.o.a.

	**50 μL**	**160 μL**	**500 μL**	$\%\;\text{o}\text{f}\;\text{i}nhibitio\text{n}=\frac{\left(D_{o}-D_{x}\right)}{D_{o}}\times 100$			**IC**_****50****_	
							**μg/mL**	**μmol/mL**
**1**	8.7	8.7	8.7	0.00	0.00	0.00	–	–
**2**	6.7	0	0	22.99	100	100	30.4 ± 0	150.31 ± 0
**3**	8.7	4	0	0.00	54.02	100	–	–
**4**	6.5	2.2	0	25.29	74.71	100	54.4 ± 0	214.10 ± 0
**5**	3.8	1.8	0.3	56.32	79.31	96.55	18.8 ± 0	74.28 ± 0
**6**	8	1.8	0	8.05	79.31	100	–	–
**7**	8.7	5.2	0	0.00	40.23	100	–	–
**8**	8.7	5.4	0	0.00	37.93	100	–	–
**9**	8.7	8.7	0	0.00	0.00	100	–	–
**10**	8.7	8.7	0	0.00	0.00	100	–	–
**11**	8.7	6.2	1.2	0.00	28.74	86.21	–	–
**12**	8.7	5.6	0	0.00	35.63	100	–	–

Table 6 shows the three best anti-*Fusarium* candidates IC_50_ values, where the highest value is for ligand **5** having IC_50_ = 18.8 μg/mL and IC_50_ = 74.28 μmol/L, substituted pyridine ring with Brome.

**Table 6:** The MIC, pIC_50_, and LogP values of the studied ligands **2**, **4**, and **5**.

	**IC_**50**_ (μmol/L)**	**pIC_**50**_**	**LogP**
**2**	150.31	2.18	1.49
**4**	214.10	2.33	1.24
**5**	74.28	1.87	1.93

The lipophilicity (Veber et al., 2002; Leeson and Springthorpe, 2007; Podunavac-Kuzmanovic et al., 2008; Mannhold et al., 2009; Arnott and Planey, 2012; Hadda et al., 2014; Sima et al., 2017) characteristic of a ligand is the most important key in drug design and discovery, contributing to the ADMET-Tox (Gleeson et al., 2011; Glaab, 2016; Kauthale et al., 2017; Dhandapani and Balachandar, 2019) (administration, distribution, metabolism, excretion, and toxicity) of drugs and providing us insights about their solubility, cell membrane permeability, and so on (Arnott and Planey, 2012). It is expressed as the LogP of a compound with two different solvents: 1-octanol showing the lipid membrane and water as the model for cytoplasm (Holladay, 2015).

**Figure 8:**
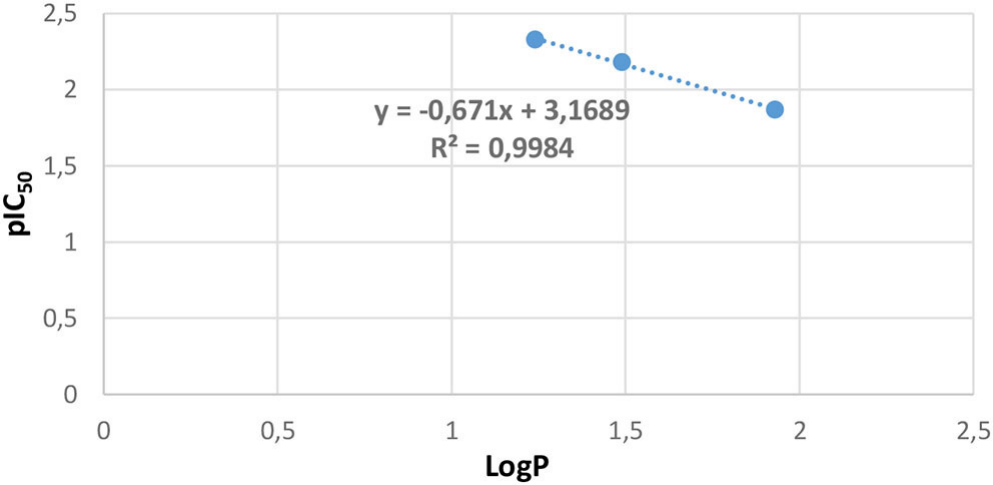
Lipophilicity and antifungal activity plot.

In our study, the LogP values of compounds **2**, **4**, and **5** were calculated handling the “Marvin sketch 19.13” software (https://chemaxon.com/products/marvin). The results are summed up in Table 6, which demonstrates that ligand **5** is the most effective with the biggest value of lipophilicity (LogP = 1.93); the order of inhibition efficiency IC_50_ correlated well with the following lipophilicity values order: **5** > **2** > **4**. The LogP values were in the optimum region (LogP = 3), and hence, all three ligands could pass through the lipid membrane by the intense decrease in their IC_50_ values; for instance, for ligand **4**, IC_50_ = 214.10 μmol/L and LogP = 1.24. Based on these results, lipophilicity and antifungal activity correlation plot are represented in Figure 8, and the analysis of these results established a notable linear correlation between pIC_50_ and LogP according to the following equation: *y* = −0.671*x* + 3.1689, where *x*: LogP, *y*: pIC_50_, and correlation coefficient of *R*^2^ = 0.9984.

To sum up, the results proved a correlation between the lipophilicity and antifungal activity of the ligands, but further precise experiments recommended to figure out the unfamiliar mechanism of action for the investigated ligands.

### Theoretical Investigations

#### DFT Calculations

MEP may be a truly capable tool utilized to calculate or envision the reactive zones of nucleophilic and electrophilic attacks on a molecular system. It is often generated by mapping the ESP onto the isoelectron density surface of the molecule, providing us the chance to know the distribution of the electronic charge all over the structure. MEP mapping remains helpful in figuring out the synergy of a molecule with its environment and monitoring the hydrogen binding interactions, for its biological recognition processes (Kryachko, 2013; Alpaslan et al., 2019).

**Figure 9:**
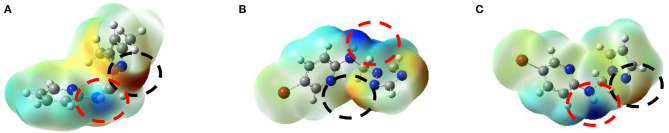
MEP surfaces for the three best anti-*Fusarium* candidates **2** **(A)**, **4** **(B)**, and **5** **(C)**, where the negative regions (black circle) are related to electrophilic reactivity, whereas the positive regions (red circle) are for nucleophilic reactivity.

In our research, the optimized structures are in their global minima because of its positive frequencies values, MEP maps of compounds **2**, **4**, and ***5*** generated based on their density functional theory (DFT) optimized geometries; displayed in Figure 9. The negative ESP regions of compound **2** mainly concentrated over the nitrogen atoms of the pyrazole ring with a value of −1.174 eV, whereas the negative charges of compound **4** were situated on the nitrogen atoms of the triazole ring with a value of −1.612 eV. Compound **5** has negative charges located on the nitrogen atoms of the pyrazole ring with a value of −0.492 eV greater than the negative charge of compound **9** reported by Tighadouini et al. (2019), which has −1.850 eV. The positive charges of three ligands **2**, **4**, and **5** located were in the NH region of the N-C-N junction with values of 0.989, 1.612, and 1.324 eV, respectively. These results permitted us valuable information on the potential sites engaged in interactions between hydrogen bonds and the amino acid residues of protein receptors.

#### Blind Docking/Virtual Screening

**Figure 10:**
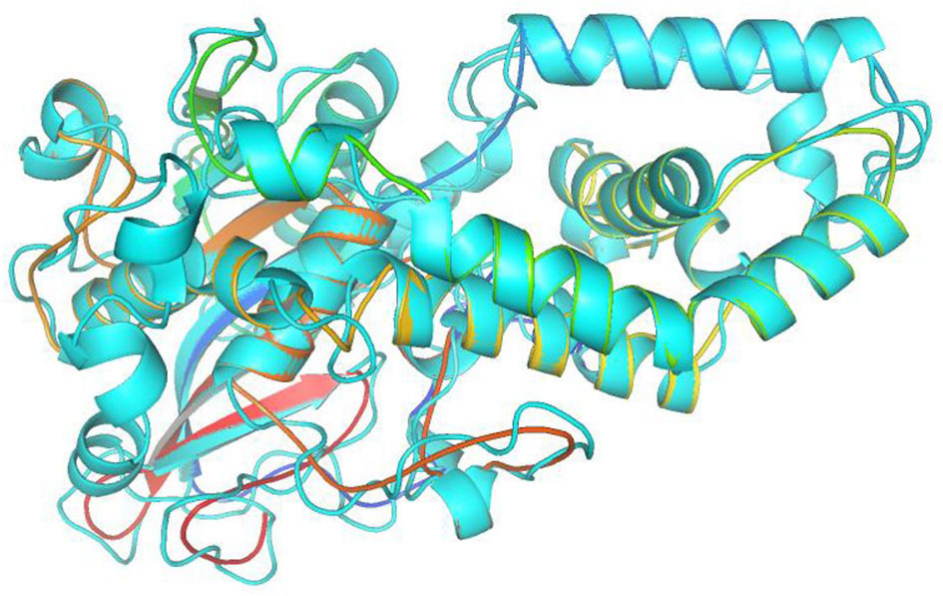
Structure alignment of homology modeled Fophy protein (colored) and its template 3K4P (blue).

In this study, the rotatable bonds of the ligand flexibility were allowed, while the protein was adopted as a rigid structure. As a first step of this study, the three-dimensional structures of the homology modeled Fophy protein and its template, which is *Aspergillus niger* phytase (PDB:3K4P) (Gontia-Mishra et al., 2014; Toubi et al., 2019), were aligned using Pymol software (Seeliger and de Groot, 2010), and it is presented in Figure 10.

As presented, there is good alignment between the two structures with smooth change; forward blind docking/virtual screening in both structures is provided using the active compounds against F.o.a., where the dataset compounds are the ones in Table 7.

**Table 7:** pKi, pMIC, pIC50, and ADMET properties of all the dataset compounds.

**ID**	**Affinity (kcal/mol)**	**pMIC**	**pIC_**50**_**	**MW (<500 Da)**	**HAC (<10)**	**HDO (<5)**	**nRot (<10)**	**TPSA**	**logP (<5)**	**nVio**
Atrazine	−5.8	–	–	215.68	3	2	4	62.73	1.74	No
Linuron	−5.8	–	–	249.09	2	1	4	41.57	2.59	No
Diuron	−5.5	–	–	233.09	1	1	3	32.34	2.54	No
Crotoxyphos	−6.4	–	–	314.27	6	0	8	80.87	2.69	No
Dichlorvos	−4.3	–	–	220.98	4	0	4	54.57	1.60	No
Phosphamidon	−5.6	6.82	–	299.69	5	0	8	74.88	1.68	No
Monensin-H	−6.8	5.66	–	670.88	11	4	10	153.37	3.63	Yes
Lasalocid	−7.4	–	–	590.80	8	4	13	133.52	5.12	Yes
Benomyl	−6.3	–	–	290.32	4	2	8	85.25	1.85	No
1	−6.5	–	–	219.20	5	2	4	92.93	−0.06	No
2	−5.9	–	3.82	202.26	2	1	3	42.74	1.57	No
3	−6.2	–	–	216.29	2	1	3	42.74	2.01	No
4	−5.8	–	3.67	254.09	3	1	3	55.63	1.29	No
5	−6.0	–	4.13	253.10	2	1	3	42.74	1.70	No
6	−6.4	–	–	281.16	2	1	3	42.74	2.33	No
7	−5.0	–	–	180.23	2	1	3	70.98	1.04	No
8	−5.6	–	–	208.28	2	1	3	70.98	1.72	No
9	−6.2	–	–	202.26	2	1	3	42.74	1.45	No
10	−5.3	–	–	234.26	4	0	5	61.94	1.07	No
11	−5.6	–	–	204.23	3	2	3	62.97	0.85	No
12	−6.3	–	–	232.29	3	2	3	62.97	1.50	No
13	−6.3	–	–	216.29	2	1	3	42.74	1.98	No
14	−6.5	–	2.60	247.25	4	1	4	88.56	1.18	No
15	−6.2	–	3.39	295.18	2	1	3	42.74	2.62	No
16	−6.0	–	3.48	360.05	2	1	3	42.74	2.92	No
17	−6.7	–	2.59	235.24	5	3	3	96.09	0.62	No
18	−6.8	–	2.60	233.27	4	2	3	75.86	1.25	No
19	−7.0	–	2.91	246.27	4	2	4	80.04	1.06	No
20	−6.3	–	3.16	229.32	5	1	5	29.85	2.48	No
21	−4.8	—-	2.54	179.22	2	1	4	46.92	1.01	No
22	−6.0	–	2.63	215.29	2	1	4	29.85	2.20	No
23	−5.5	–	2.59	188.23	2	1	3	42.74	1.30	No
24	−6.2	–	2.56	203.24	3	1	3	55.63	1.14	No
25	−7.1	–	2.58	243.30	2	1	4	46.92	2.29	No
26	−7.3	–	4.06	277.36	1	0	4	21.06	3.68	No
27	−6.7	–	3.70	249.31	1	0	4	21.06	2.99	No
28	−6.4	–	3.18	229.32	2	1	4	29.85	2.48	No
29	−6.3	–	–	217.22	3	2	4	67.15	0.92	No
30	−6.0	1.55	–	305.38	5	0	7	65.18	1.62	No
31	−5.9	1.57	–	319.40	5	0	8	65.18	1.91	No
32	−7.0	1.74	–	409.52	5	0	10	65.18	3.41	No
33	−6.2	1.66	–	409.52	5	0	10	65.18	3.41	No
34	−6.3	1.70	–	319.40	5	0	7	65.18	1.87	No
35	−6.0	1.70	–	347.46	5	0	8	65.18	2.53	No
36	−6.9	2.52	–	434.53	5	1	9	80.97	3.22	No
37	−6.3	2.60	–	310.40	3	0	5	51.77	2.45	No
38	−7.2	2.30	–	426.47	7	0	11	104.37	2.52	No
39	−6.9	2.60	–	309.41	2	0	5	38.88	3.05	No
40	−6.2	1.40	–	425.48	6	0	11	91.48	3.15	No
41	−7.3	2.60	–	354.41	4	0	6	84.70	2.31	No
42	−6.5	2.30	–	470.48	8	0	12	137.30	2.46	Yes
43	−6.4	1.40	–	323.44	2	0	5	38.88	3.28	No
44	−6.7	1.10	–	439.51	6	0	11	91.48	3.30	No
45	−7.8	2.00	–	302.42	4	0	4	42.12	1.66	No
46	−6.4	2.30	–	418.49	8	0	10	94.72	1.74	No
47	−5.8	2.00	–	304.43	4	0	7	42.12	1.93	No
48	−5.9	1.70	–	420.51	8	0	13	94.72	2.00	No

From Table 7, only 31 compounds have pMIC values between 2.54 and 7.10, with 42 having pIC_50_ values between 1.09 and 4.69; thus, there were 73 compounds in dataset of 48 compounds other than the nine first compounds as bactericides and insecticides. The pKi values were from 2.74 and 5.83, with 97% of the compounds not violating the Lipinski's rule of 5, which makes it a good database for future *in vivo* tests.

**Figure 11:**
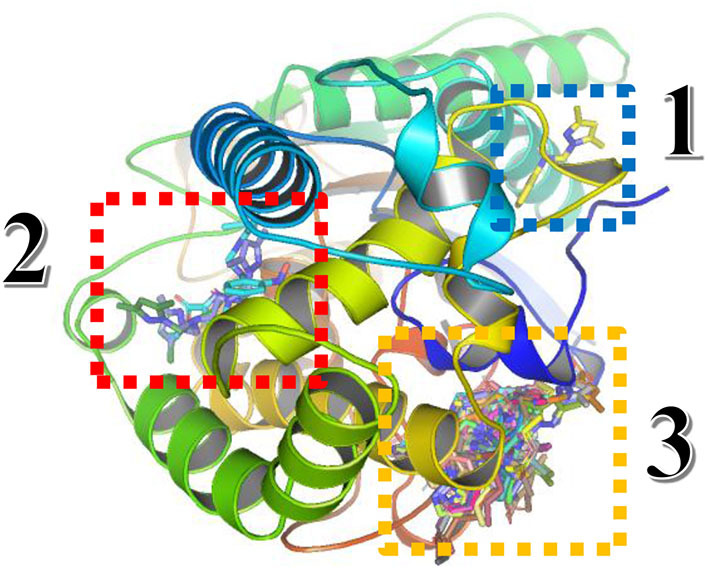
Three-dimensional presentation of the three binding sites found by Blind docking/virtual screening.

For the Blind docking with virtual screening, the results obtained from Autodock Vina are presented in Figure 11 and Table 8.

**Table 8:** Binding modes residues data for each ligand studied.

**Site**	**ID**	**SER77**	**GLU78**	**HIS81**	**LEU82**	**PHE90**	**SER91**	**LEU92**	**LYS95**	**PHE253**	**ALA259**	**ASP260**	**HIS322**	**ILE326**	**ASP329**	**TYR330**	**SER333**	**HIS340**	**ASN398**	**ALA421**	**GLU422**	**ASN423**	**ILE424**	**THR425**	**THR427**	**PHE430**	**SER431**	**TRP434**
3	Atrazine	–	–	y	y	y	–	–	–	–	–	–	–	–	y	y	–	–	–	–	–	–	y	–	–	–	y	y
	Linuron	–	–	y	–	–	–	–	y	–	–	–	–	–	y	–	y	–	–	–	y	–	–	–	–	–	y	y
	Diuron	–	–	y	–	y	–	–	y	–	–	–	–	–	–	–	–	–	–	–	y	–	–	–	–	–	y	y
	Crotoxyphos	–	–	–	–	–	–	–	–	–	–	–	–	y	–	y	–	–	–	–	y	–	–	–	–	–	–	y
	Dichlorvos	–	–	–	y	–	–	–	–	–	–	–	–	–	y	–	–	–	–	–	–	–	y	–	–	–	–	y
	Phosphamidon	–	–	–	–	–	–	–	–	–	–	–	–	–	y	–	–	–	–	–	–	–	–	–	–	–	y	y
	2	–	–	–	y	–	–	–	–	–	–	–	–	–	y	y	–	–	–	–	–	–	y	–	–	–	–	y
	4	–	–	y	y	–	–	–	–	–	–	–	–	–	y	–	–	–	–	–	–	–	y	–	–	–	–	y
	5	–	–	–	y	–	–	–	–	–	–	–	–	–	y	–	–	–	–	–	–	–	y	–	–	y	y	–
	14	–	–	–	y	–	–	–	–	–	–	–	–	–	y	–	–	–	–	–	–	–	y	–	–	–	–	y
	15	–	–	y	y	–	–	–	–	–	–	–	–	y	y	y	–	–	–	–	y	–	y	–	–	–	–	y
	16	–	–	y	–	y	–	–	–	–	–	–	–	–	y	–	–	–	–	–	–	–	y	–	–	–	–	y
	17	–	–		y	–	–	–	–	–	–	–	–	–	y	–	–	–	–	–	y	–	y	–	–	–	–	y
	18	–	–	y	y	–	–	–	–	–	–	–	–	y	y	y	–	–	–	–	y	–	y	–	–	–	y	y
	19	–	–	–	y	–	–	–	–	–	–	–	y	–	y	–	–	–	–	–	y	y	y	–	y	–	–	–
	20	–	–	–	y	–	–	–	–	–	–	–	–	y	y	–	–	–	–	–	–	–	y	–	–	–	–	y
	21	–	–	–	–	–	–	–	–	–	–	–	–	–	–	–	–	–	–	–	–	–	y	–	–	–	–	–
	22	–	–	–	–	–	–	–	–	–	–	–	–	–	y	y	–	–	–	–	–	–	y	–	–	–	–	y
	23	–	–	–	–	–	–	–	–	–	–	–	–	y	y	–	–	–	–	–	–	–	y	–	–	–	–	–
	24	–	–	y	y	–	–	–	–	–	–	–	–	–	y	y	–	–	–	–	–	–	y	–	–	–	–	–
	25	–	–	–	y	–	–	–	–	–	–	–	–	y	–	y	–	–	–	–	–	y	y	–	–	–	y	–
	26	–	–	–	–	–	–	–	–	–	–	–	–	y	–	–	–	–	–	y	y	y	y	–	–	–	–	–
	27	–	–	–	–	–	–	–	–	–	–	–	–	y	y	–	–	–	–	y	–	–	y	–	–	–	–	–
	28	–	–	y	y	y	–	–	–	–	–	–	y	–	y	–	–	–	–	–	–	–	–	–	–	–	–	y
	30	–	–	y	y	–	–	–	–	–	–	–	–	–	y	–	–	–	–	–	–	–	y	–	–	–	–	–
	31	–	–	–	–	–	–	–	–	–	–	–	–	–	y	y	–	–	–	–	–	–	y	–	–	–	–	y
	32	–	y	–	–	–	–	–	–	–	–	–	y	y	–	–	–	–	–				y	–	–	–		y
	33	–	–	–	–	–	–	–	–	y	y	y	–	–	–	–	–	y	y	–	–	–	–	–	–	–	–	–
	34	–	–	–	–	–	–	–	–	–	–	–	–	–	y	y	–	–	–	–	–	–	y	–	–	–	y	–
	35	–	–	–	y	–	–	–	–	–	–	–	y	–	–	–	–	–	–	–	y	–	–	–	–	–	y	–
	36	–	–	–	–	–	–	–	–	–	–	–	–	y	y	–	–	–	–	–	–	y	y	–	–	–	–	y
	37	–	–	–	–	–	–	–	–	–	–	–	–	y	y	–	–	–	–	–	–	–	y	–	–	–	–	–
	38	–	–	–	–	–	–	–	–	–	–	–	–	y	y	–	–	–	–	y	–	y	y	–	–	–	–	y
	39	–	–	–	–	–	–	–	–	–	–	–	–	y	y	–	–	–	–	y	–	–	y	–	–	–	–	–
	40	–	–	–	–	–	–	–	–	–	–	–	–	y	y	–	–	–	–	–	–	y	y	y	–	–	–	–
	41	–	–	–	y	–	–	–	–	–	–	–	–	–	y	–	–	–	–	–	–	y	y	–	–	–	y	–
	42	–	–	y	–	y	–	y	y	–	–	–	–	–	y	–	–	–	–	–	–	–	y	–	–	–	–	y
	43	–	–	y	y	y	–	–	y	–	–	–	y	–	y	–	–	–	–	–	–	–	–	–	–	–	–	y
	44	–	–	y	y	y	–	y	y	–	–	–	–	y	y	y	–	–	–	–	–	–	–	–	–	–	–	–
	45	–	–	y	–	–	y	–	y	–	–	–	–	–	–	y	–	–	–	–	–	–	y	–	–	–	–	–
	46	y	–	y	y	–	y	–	y	–	–	–	–	y	–	–	–	–	–	–	–	–	y	–	–	–	y	–

“*y” means that there is binding between ligand and target*.

From the results above, the majority of the ligands screened are binding into the third active site, whereas only compound **3** is in the first site and the compounds monensin-H; **33**, **54**, and **89** are in the second one, but with which residues the compounds are interacting in the third site; thus, the following data were collected as shown in Table 8.

From data above, the site contains the following residues: SER77, GLU78, HIS81, LEU82, PHE90, SER91, LEU92, LYS95, PHE253, ALA259, ASP260, HIS322, ILE326, ASP329, TYR330, SER333, HIS340, ASN398, ALA421, GLU422, ASN423, ILE424, THR425, THR427, PHE430, SER431, and TRP434, where 15.09% of the bonds are with ILE424, 14.62% with ASP329, 10.37% with TRP434, 9.43% with LEU82, and 7.5% with ILE326.

**Figure 12:**
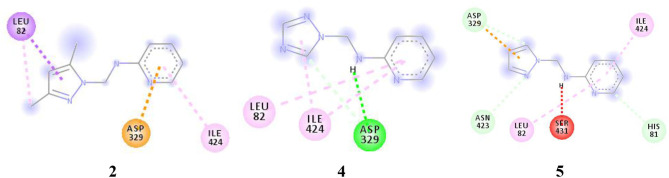
The two-dimensional interactions of ligand **2**, **4**, and **5** with the chosen active site in the Fophy protein.

For more specific study, the modes of binding interactions for ligands 2, 4, and 5, which are in the third site of the Fophy protein, are presented in Figure 12. Based on the docking results, ligand 5 reached the strongest affinity of −6.0 kcal/mol, whereas ligand 2 showed −5.9 kcal/mol, and ligand 4 showed −5.8 kcal/mol.

For more specific study, the modes of binding interactions for ligands **2**, **4**, and **5**, which are in the third site of the Fophy protein, are presented in Figure 12. Based on the docking results, ligand **5** reached the strongest affinity of −6.0 kcal/mol, whereas ligand **2** showed −5.9 kcal/mol, and ligand **4** showed −5.8 kcal/mol.

As presented in Figure 12 and Table 9, the studied compounds have multiple bonds, but only compounds **5** and **4** have carbon hydrogen bonds, whereas the strongest stay for the compounds with distance ranges from 1.59 to 5.40 Å for all bonds.

**Table 9:** The binding interactions between the best-studied ligands and the Fophy protein selected active site.

	**Affinity (kcal/mol)**	**Interaction (L-AA)**	**Distance (Å)**
**2**	−5.9	Pyrazole-LEU82: **pi-sigma** Pyrazole-LEU82: **pi-alkyl** Pyridine-ASP329: **pi-anion** Pyridine-ILE424: **alkyl**	3.74 5.05 3.54 5.41
**4**	−5.8	Pyrazole-ILE424: **pi-alkyl** Pyrazole-ASP329: **carbon hydrogen bond** NH-ASP329: **conventional hydrogen bond** Pyridine-LEU82: **pi-alkyl** Pyridine-ILE424: **pi-alkyl**	5.24 3.15 2.03 5.44 5.13
**5**	−6.0	Pyrazole-ASP329: **carbon hydrogen bond** Pyrazole-ASN423: **carbon hydrogen bond** Pyridine-HIS81: **carbon hydrogen bond** Pyrazole-ASP329: **pi-anion** Pyridine-LEU82: **pi-alkyl** Pyridine-ILE424: **pi-alkyl** NH-SER431: **unfavorable donor**	3.20 3.64 3.60 3.30 5.40 5.13 1.59

By comparing the results of the docking studies for the Fophy protein, it is found that compound **5** has the best affinity followed by compounds **2** and **4**, so there is good agreement with the experimental results where their IC_50_ values are in the following order: 74.28 (5) < 150 (2) < 214.10 (4), but needs more investigations with much more compounds to build a model for phytase inhibitor prediction.

**Figure 13:**
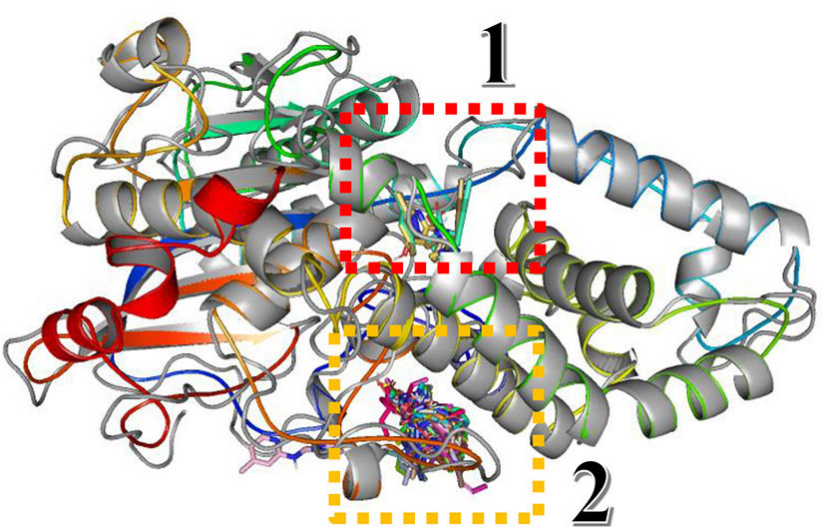
Structure alignment of Blind docking/virtual screening results on *Aspergillus niger* phytase protein and Fophy.

For protocol validation, blind docking/virtual screening was done for the same ligands and parameters with *A. niger* phytase (PDB:3K4P), which is the homology modeling template of the studied Fophy protein, implemented in Autodock Vina, and the results are presented in Figure 13.

**Figure 14:**
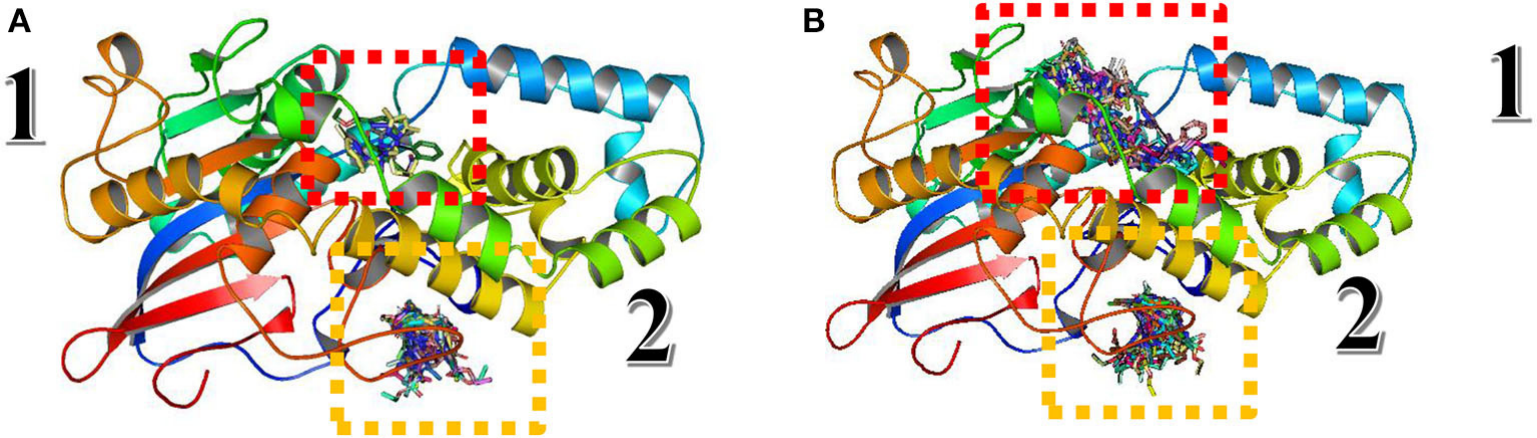
Blind docking/virtual screening of Fophy protein using Autodock Vina **(A)** and Dockthor **(B)**.

As presented in Figure 13, there are two different sites instead of three found in Fophy protein; compound 3 in the third one is eliminated because it is not active against F.o.a., and commonly most of the ligands screened are in the same site (the third one for Fophy protein and the second for *A. niger* phytase protein), which is approved also from all ligand visualizations using Autodock Vina and Dockthor as presented in Figure 14.

## Conclusion

Series of 12 pyrazole- and triazole-based ligands were prepared in good yield up to 99.6% and characterized using ^1^H and ^13^C NMR spectroscopy. The preliminary antifungal test screening against F.o.a. proved that ligands **2**, **4**, and **5** showed close to total inhibition of the fungus with an appreciable increase in their efficiency, starting from low concentrations. This encouraged us to study their reactivity, using DFT, and binding affinity, using the Fophy protein to show ligand–protein interactions, as described in the literature. From the results obtained using computational methods such as DFT studies of MEP surfaces, we found that ligand **2** had negative potential electrostatic regions mainly concentrated over the nitrogen atoms of the pyrazole ring with a value of −1.174 eV, whereas compound **4** had negative charges on the nitrogen atoms of the triazole ring with a value of −1.612 eV. Ligand **5** had negative charges on the nitrogen atoms of the pyrazole with a value of −0.492 eV. The positive charges of three ligands **2**, **4**, and **5** were in the NH region of the N-C-N junction with values of 0.989, 1.612, and 1.324 eV, respectively. These results gave us valuable information about the potential sites involved in interactions between hydrogen bonds and the amino acid residues of the protein receptors, correlating well with the docking results. Using Blind docking/virtual screening, the predicted site contains the following residues: SER77, GLU78, HIS81, LEU82, PHE90, SER91, LEU92, LYS95, PHE253, ALA259, ASP260, HIS322, ILE326, ASP329, TYR330, SER333, HIS340, ASN398, ALA421, GLU422, ASN423, ILE424, THR425, THR427, PHE430, SER431, TRP434, where 15.09% of the bonds are with ILE424, 14.62% with ASP329, 10.37% with TRP434, 9.43% with LEU82, and 7.5% with ILE326. In the binding mode of interactions for ligands **2**, **4**, and **5**, ligand **5** reached the strongest affinity of −6.0 kcal/mol, with the strongest carbon hydrogen bonds with distance range of 1.59 to 5.40 Å for all bonds, whereas ligand **2** showed −5.9 kcal/mol, and ligand **4** showed −5.8 kcal/mol. For docking validation, the same protocol using Autodock Vina and another protocol using the Dockthor web tool give us the same predicted sites on Fophy protein.

## Data Availability Statement

The original contributions presented in the study are included in the article/Supplementary Materials, further inquiries can be directed to the corresponding authors.

## Author Contributions

YK conceived and designed the chemistry experiments, perform the theoretical investigations experiments as DFT and molecular docking, interpreted all the data, and wrote the paper. SO and RB conceived, designed, and performed the biological experiments. FA performed the homology modeling of the studied protein and revised the paper. ME, AA, NA-Z, and IW revised the manuscript. RT supervised the work and revised the manuscript. All authors contributed to the article and approved the submitted version.

## Conflict of Interest

The authors declare that the research was conducted in the absence of any commercial or financial relationships that could be construed as a potential conflict of interest.

## Abbreviations

DFT: Density Functional Theory
Fophy: *Fusarium oxysporum* phytase domain
F.o.a.: *Fusarium oxysporum f*. sp. albedinis
NMR: Nuclear Magnetic Resonance
ppm: Part per million
% inh: Percentage of inhibition
D0: Diameter in cm of F.o.a. in the control
Dx: Diameter in cm of F.o.a. in the test
MIC: Minimum Inhibition Concentration
IC50: The half maximal inhibitory concentration
LogP: Lipophilicity
ADMET-Tox, Administration, Distribution, Metabolism, Excretion: and Toxicity
MEP: Molecular Electrostatic Potential
Val: Valine
Arg: Arginine
Leu: Leucine
Glu: Glutamic acid
Lys: Lysine
His: Histidine
KBr: Potassium bromide
δ: Chemical shift
DMSO-d6: Deuterated Dimethyl sulfoxide
CDCl3: Deuterated chloroform
CD2Cl2: Deuterated dichloromethane
MeOH: Deuterated Methanol
DCM: Dichloromethane
B3LYP: 3 parameters of Becke with functional correlation gradient corrected by Lee Yang Parr.
