# Supplementary material for: Mono-Alkylated Ligands Based on Pyrazole and Triazole Derivatives Tested Against Fusarium oxysporum f. sp. albedinis: Synthesis, Characterization, DFT, and Phytase Binding Site Identification Using Blind Docking/Virtual Screening for Potent Fophy Inhibitors
Source: Front Chem. 2020 Dec 11;8:559262. doi: 10.3389/fchem.2020.559262 (PMC7759635; doi:10.3389/fchem.2020.559262)
Supplement: Supplementary file 1 [file Data_Sheet_1.PDF]

2-(((1H-1,2,4-triazol-1-yl) methyl) amino) nicotinic acid, 1

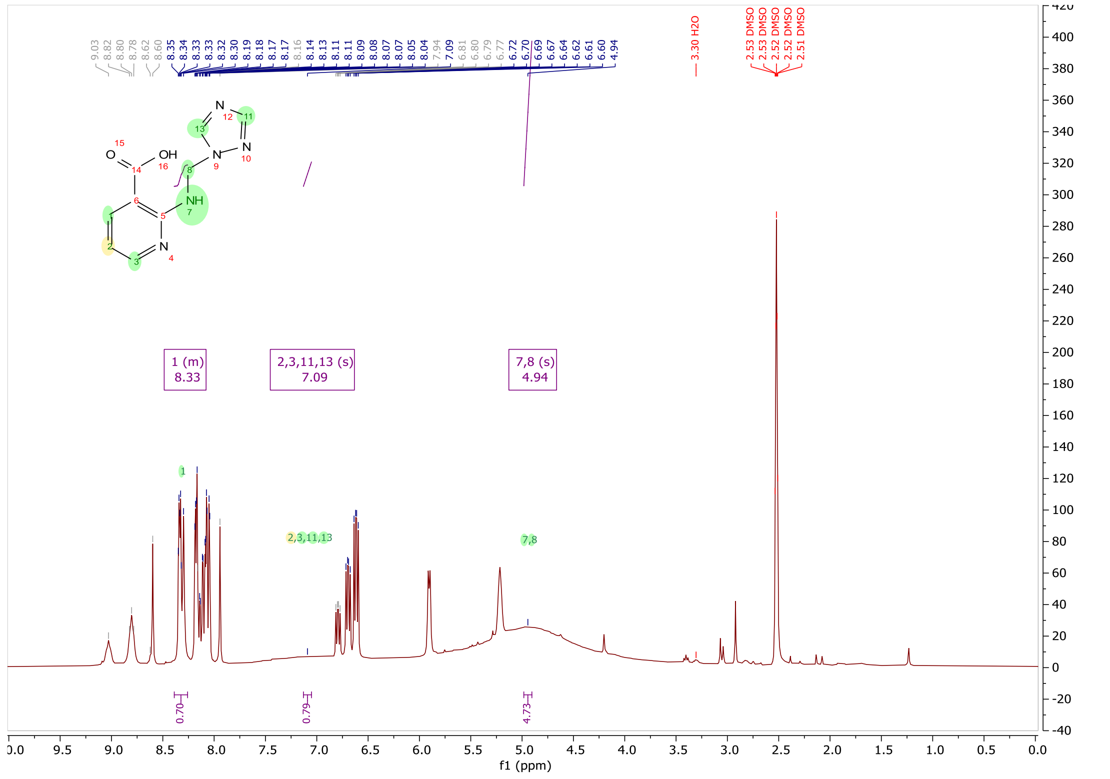

Figure 1. ^{1}H NMR spectrum of 2-(((1H-1,2,4-triazol-1-yl) methyl) amino) nicotinic acid, 1

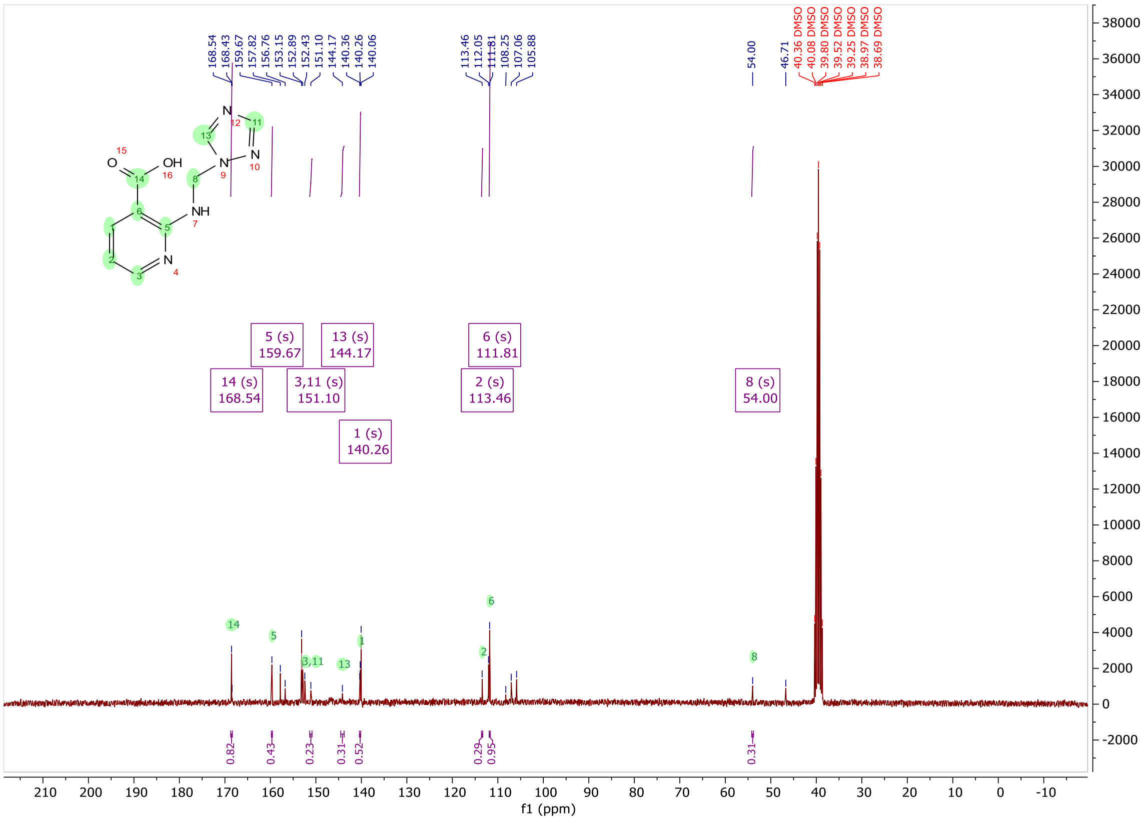

Figure 2. ^{13}C NMR spectrum of 2-(((1H-1,2,4-triazol-1-yl) methyl) amino) nicotinic acid, 1

N-((3,5-dimethyl-1H-pyrazol-1-yl) methyl) pyridin-2-amine, 2

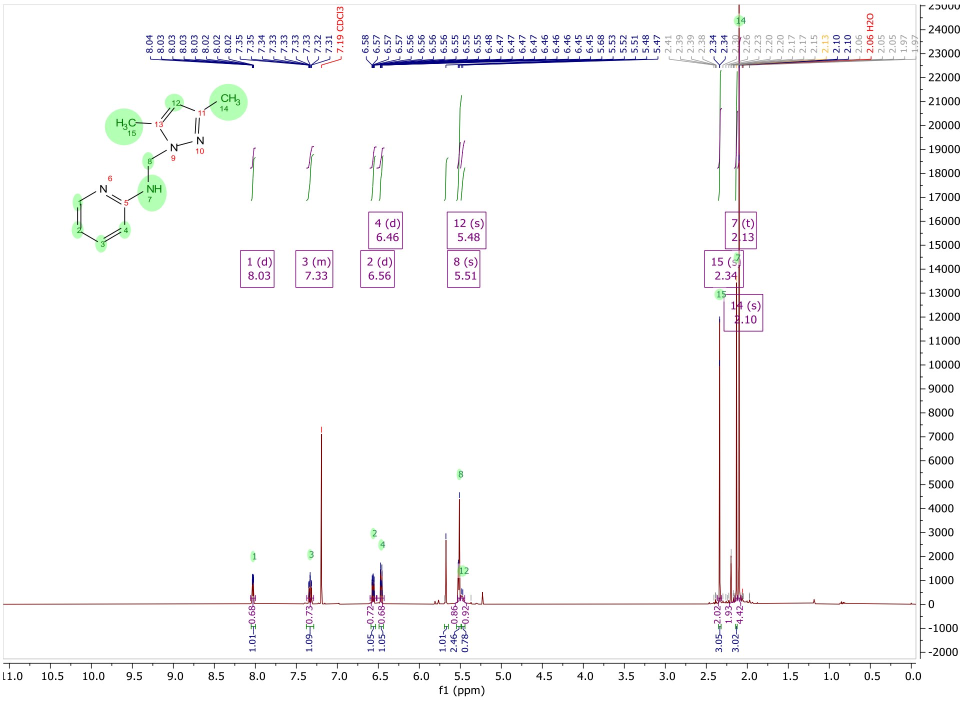

Figure 3. ^{1}H NMR spectrum of N-((3,5-dimethyl-1H-pyrazol-1-yl) methyl) pyridin-2-amine, 2

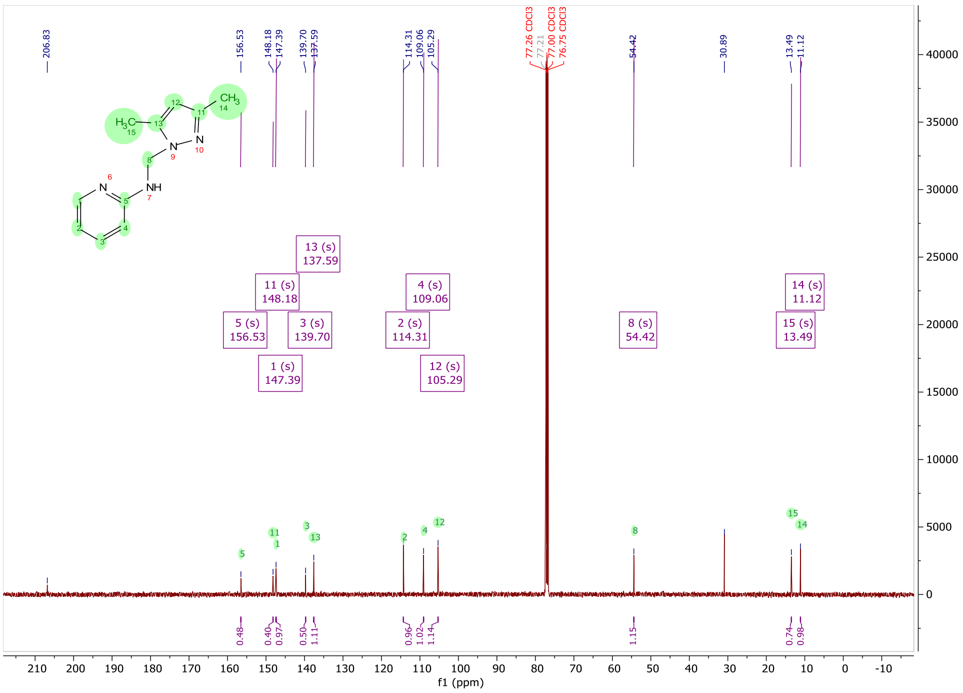

Figure 4. ^{13}C NMR spectrum of N-((3,5-dimethyl-1H-pyrazol-1-yl) methyl) pyridin-2-amine, 2

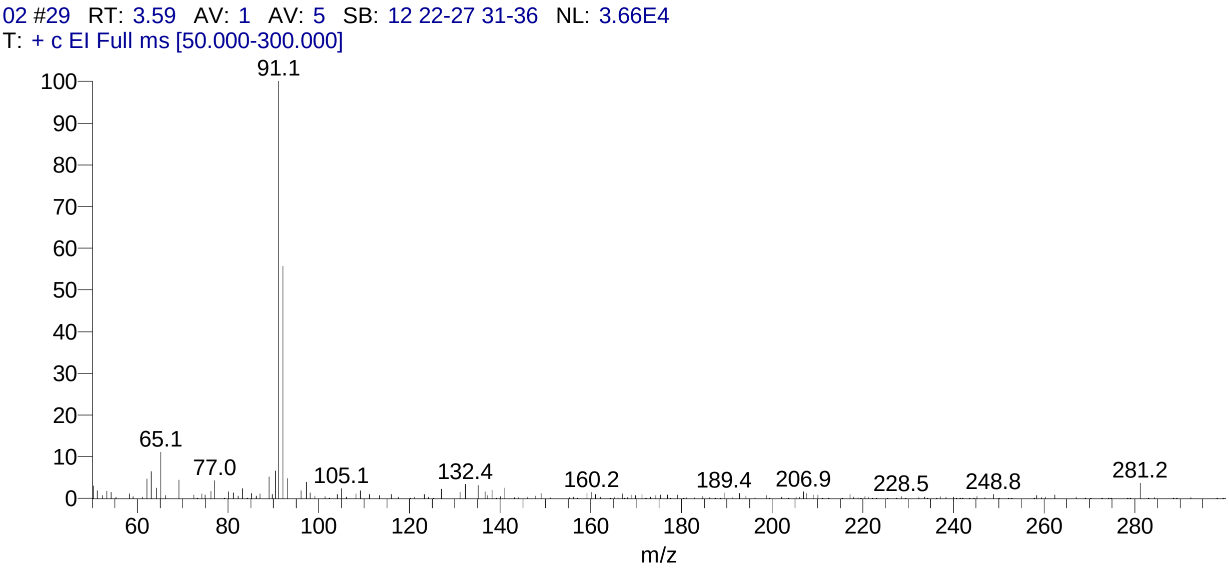

Figure 5. GC-MS spectrum of N-((3,5-dimethyl-1H-pyrazol-1-yl) methyl) pyridin-2-amine, 2

N-((3,5-dimethyl-1H-pyrazol-1-yl) methyl)-6-methylpyridin-2-amine, 3

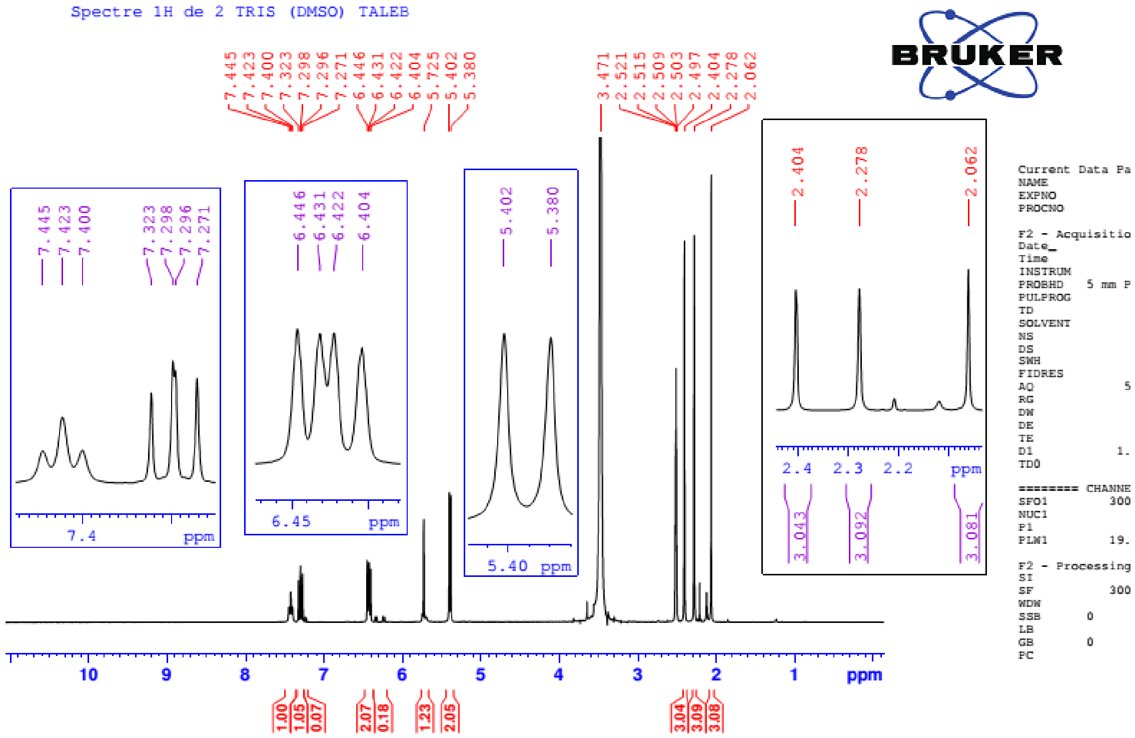

Figure 6. ^{1}H NMR spectrum of N-((3,5-dimethyl-1H-pyrazol-1-yl) methyl)-6-methylpyridin-2-amine, 3

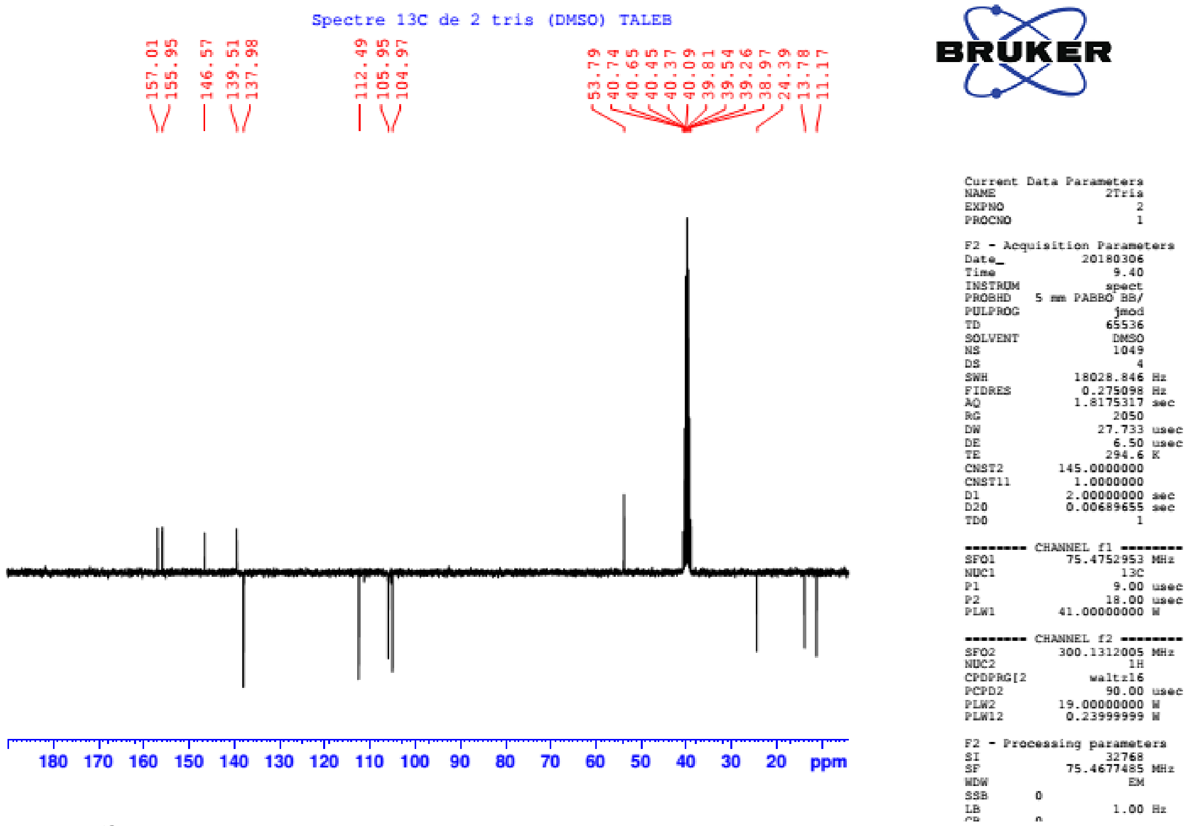

Figure 7. ^{13}C NMR spectrum of N-((3,5-dimethyl-1H-pyrazol-1-yl) methyl)-6-methylpyridin-2-amine, 3

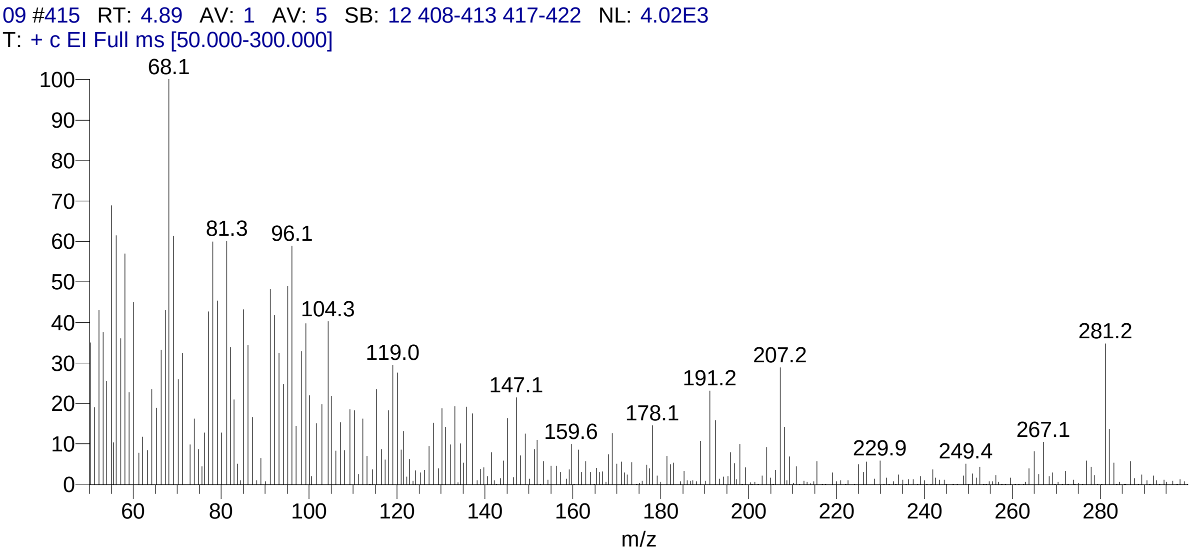

Figure 8. GC-MS spectrum of N-((3,5-dimethyl-1H-pyrazol-1-yl) methyl)-6-methylpyridin-2-amine, 3

N-((1H-1,2,4-triazol-1-yl) methyl)-5-bromopyridin-2-amine, 4

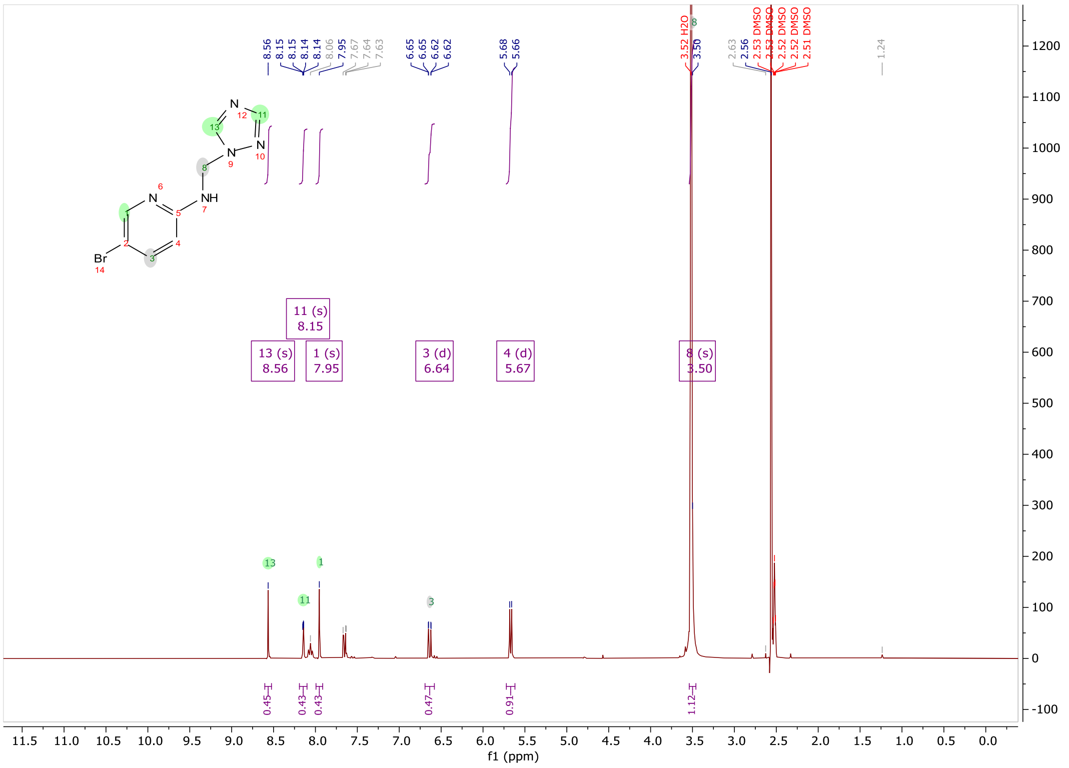

Figure 9. ^{1}H NMR spectrum of N-((1H-1,2,4-triazol-1-yl) methyl)-5-bromopyridin-2-amine, 4

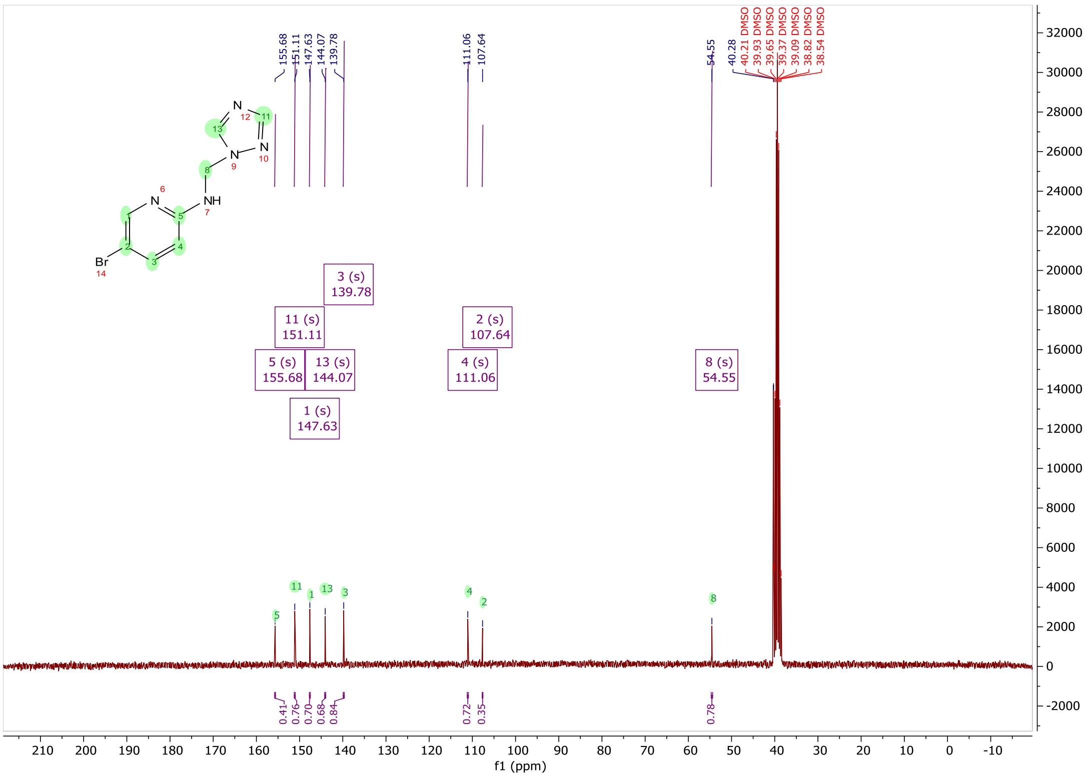

Figure 10. ^{13}C NMR spectrum of N-((1H-1,2,4-triazol-1-yl) methyl)-5-bromopyridin-2-amine, 4

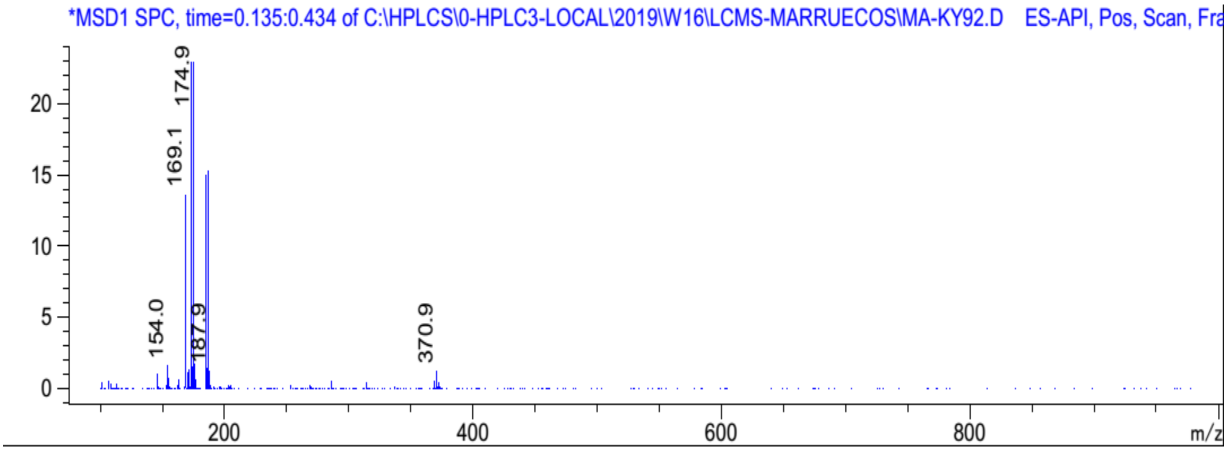

Figure 11. GC-MS spectrum of N-((1H-1,2,4-triazol-1-yl) methyl)-5-bromopyridin-2-amine, 4

N-((1H-pyrazol-1-yl) methyl)-5-bromopyridin-2-amine, 5

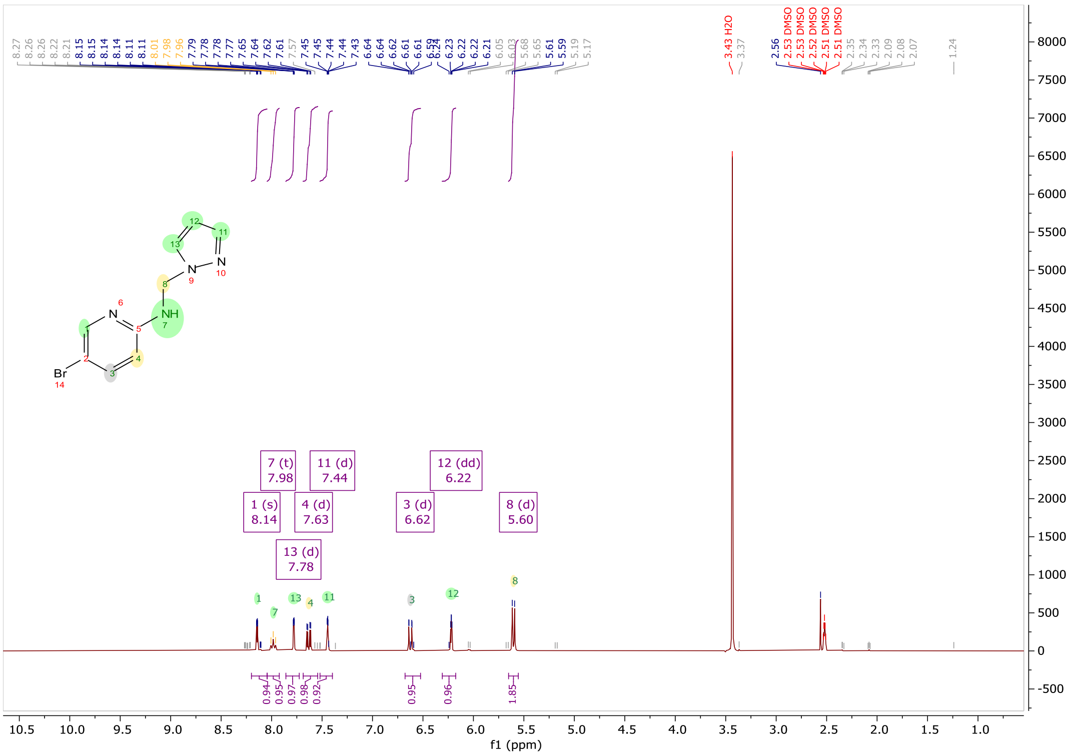

Figure 12. ^{1}H NMR spectrum of N-((1H-pyrazol-1-yl) methyl)-5-bromopyridin-2-amine, 5

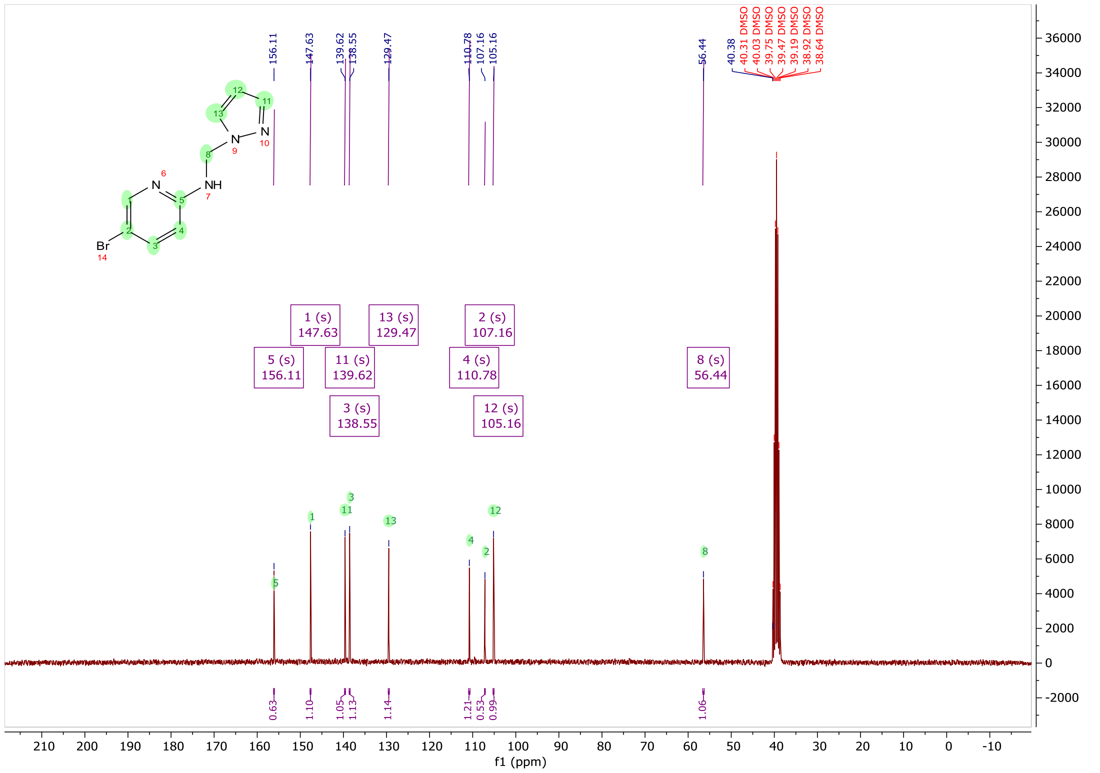

Figure 13. ^{13}C NMR spectrum of N-((1H-pyrazol-1-yl) methyl)-5-bromopyridin-2-amine, 5

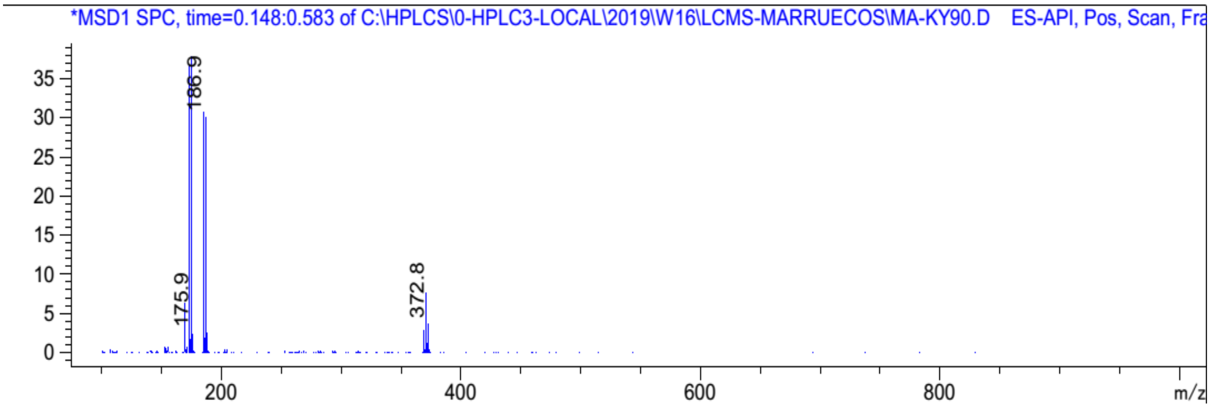

Figure 14. GC-MS spectrum of N-((1H-pyrazol-1-yl) methyl)-5-bromopyridin-2-amine, 5

5-bromo-N-((3,5-dimethyl-1H-pyrazol-1-yl) methyl) pyridin-2-amine, 6

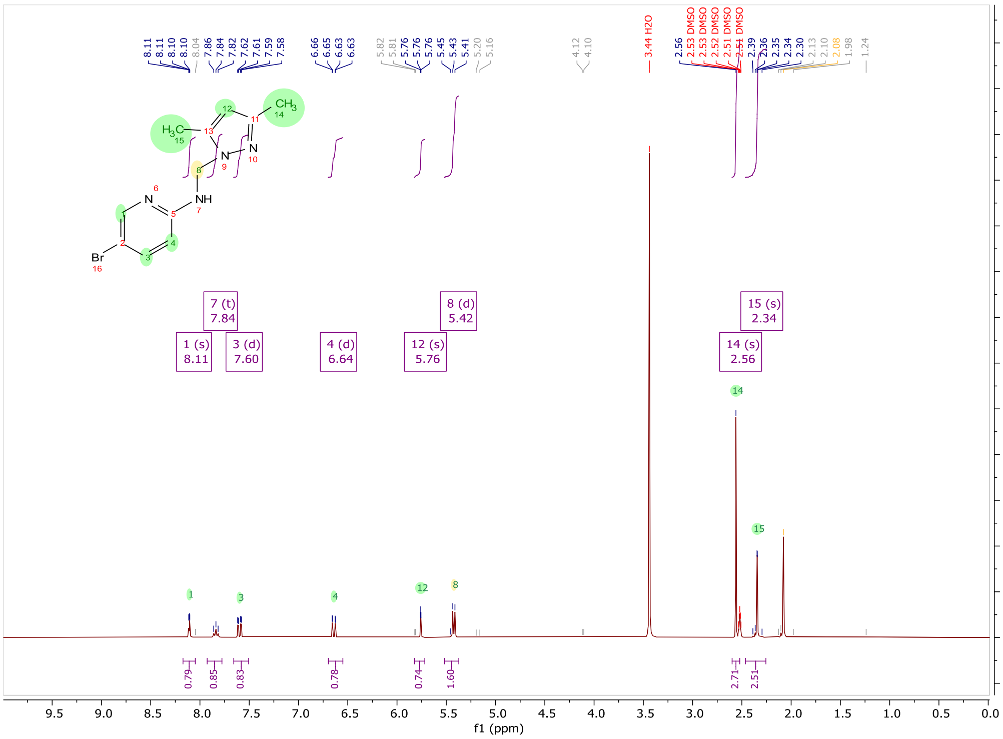

Figure 15. ^{1}H NMR spectrum of 5-bromo-N-((3,5-dimethyl-1H-pyrazol-1-yl) methyl) pyridin-2-amine, 6

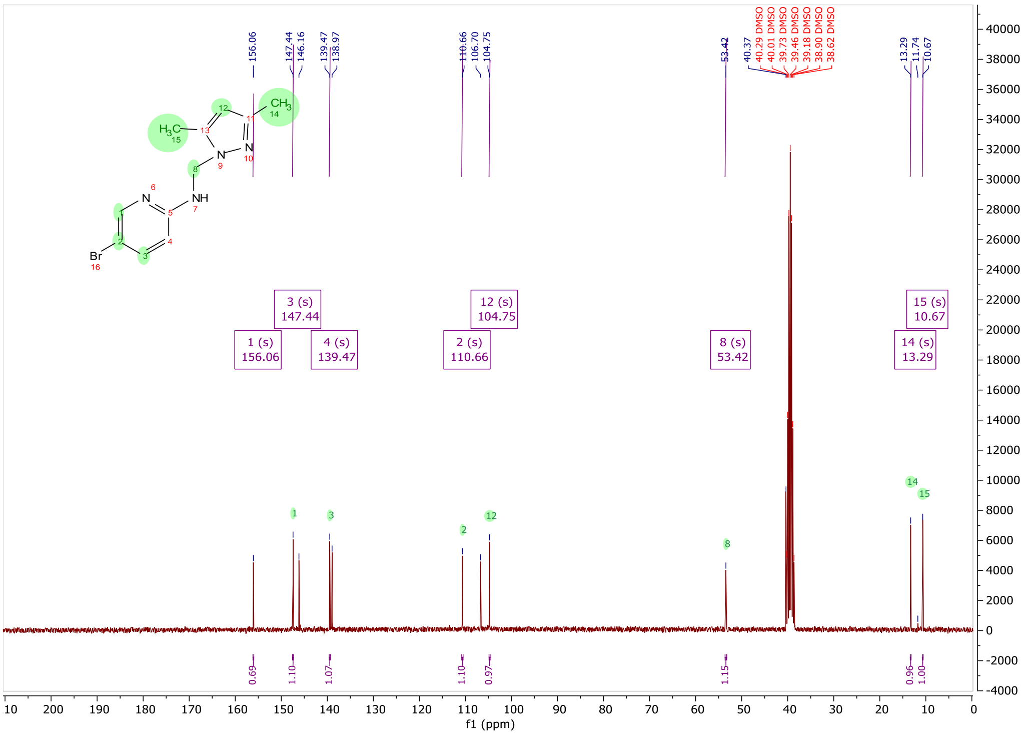

Figure 16. ^{13}C NMR spectrum of 5-bromo-N-((3,5-dimethyl-1H-pyrazol-1-yl) methyl) pyridin-2-amine, 6

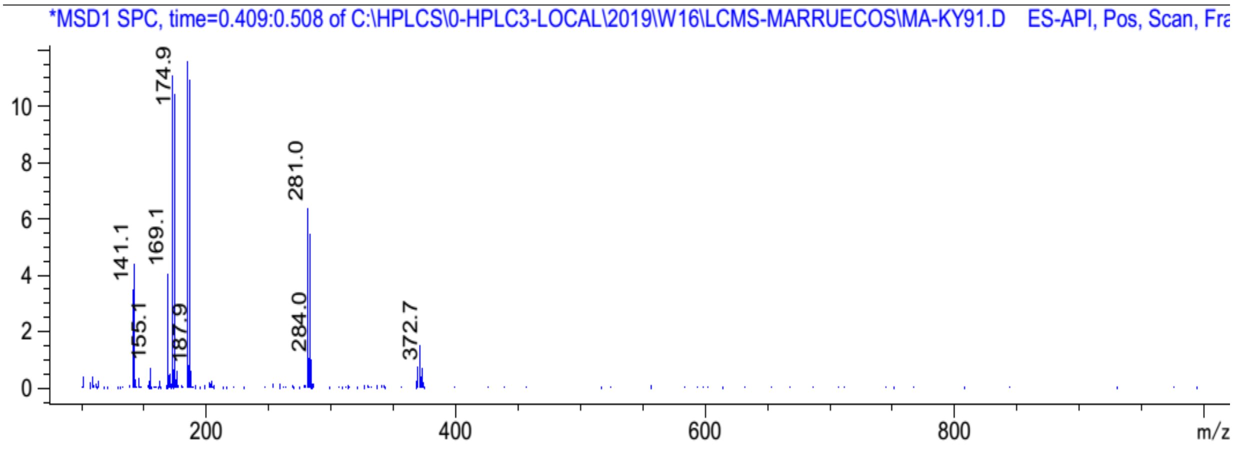

Figure 17. GC-MS spectrum of 5-bromo-N-((3,5-dimethyl-1H-pyrazol-1-yl) methyl) pyridin-2-amine, 6

N-((1H-pyrazol-1-yl) methyl) thiazol-2-amine, 7

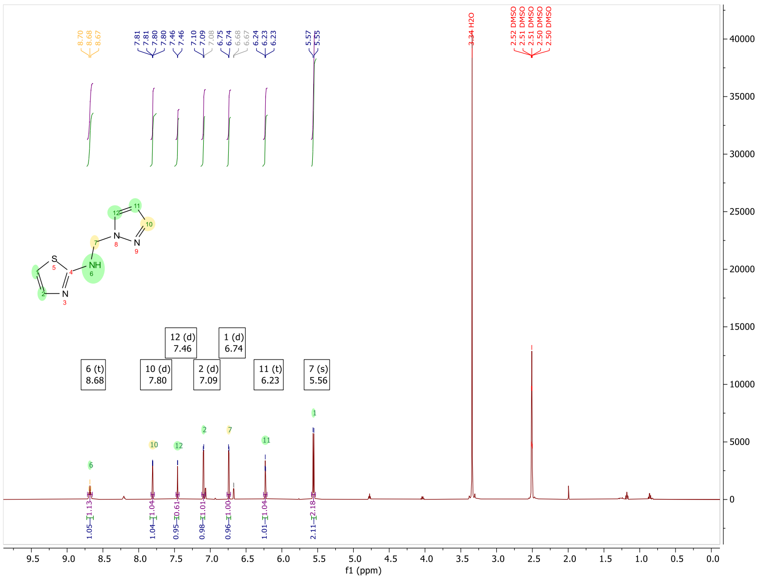

Figure 18. ^{1}H NMR spectrum of N-((1H-pyrazol-1-yl) methyl) thiazol-2-amine, 7

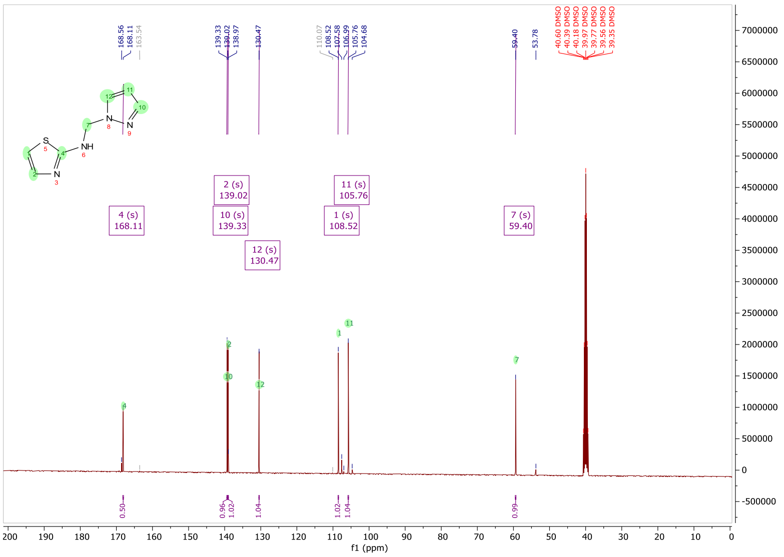

Figure 19. ^{13}C NMR spectrum of N-((1H-pyrazol-1-yl) methyl) thiazol-2-amine, 7

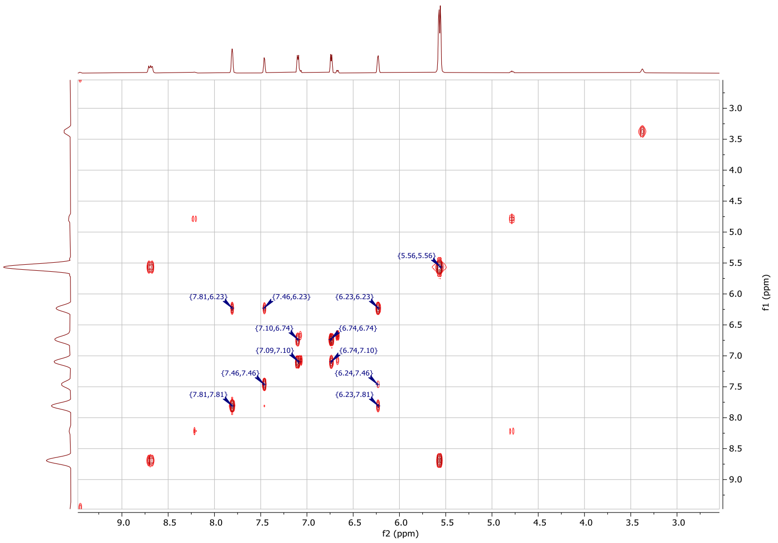

Figure 20. 2D NMR COSY spectrum of N-((1H-pyrazol-1-yl) methyl) thiazol-2-amine, 7

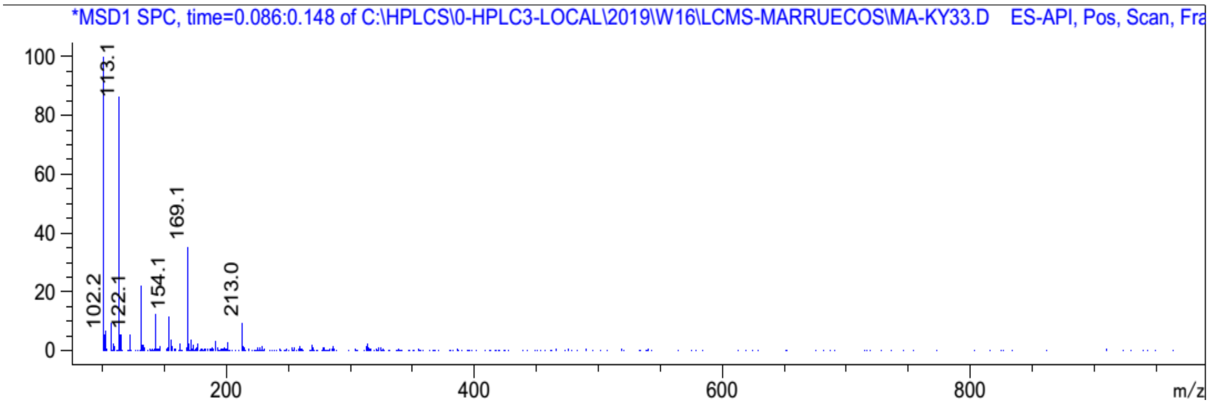

Figure 21. GC-MS spectrum of N-((1H-pyrazol-1-yl) methyl) thiazol-2-amine, 7

N-((3,5-dimethyl-1H-pyrazol-1-yl) methyl) thiazol-2-amine, 8

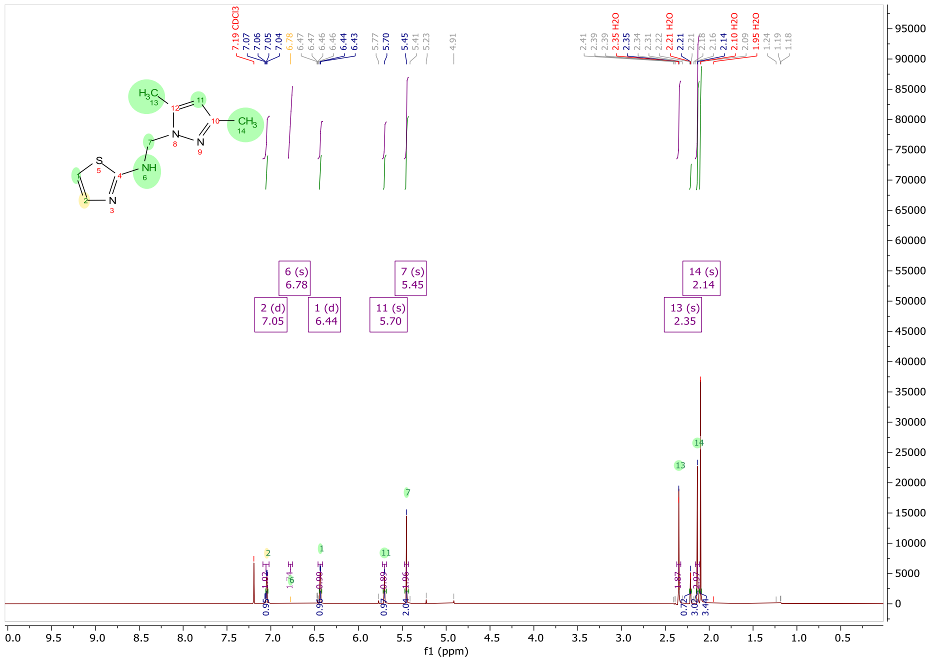

Figure 22. ^{1}H NMR spectrum of N-((3,5-dimethyl-1H-pyrazol-1-yl) methyl) thiazol-2-amine, 8

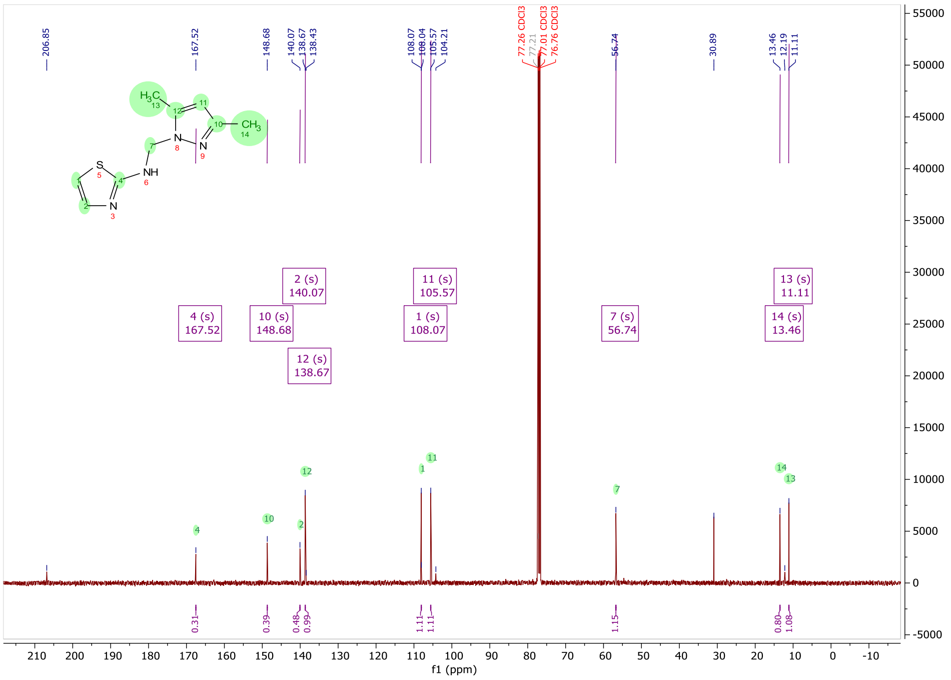

Figure 23. ^{13}C NMR spectrum of N-((3,5-dimethyl-1H-pyrazol-1-yl) methyl) thiazol-2-amine, 8

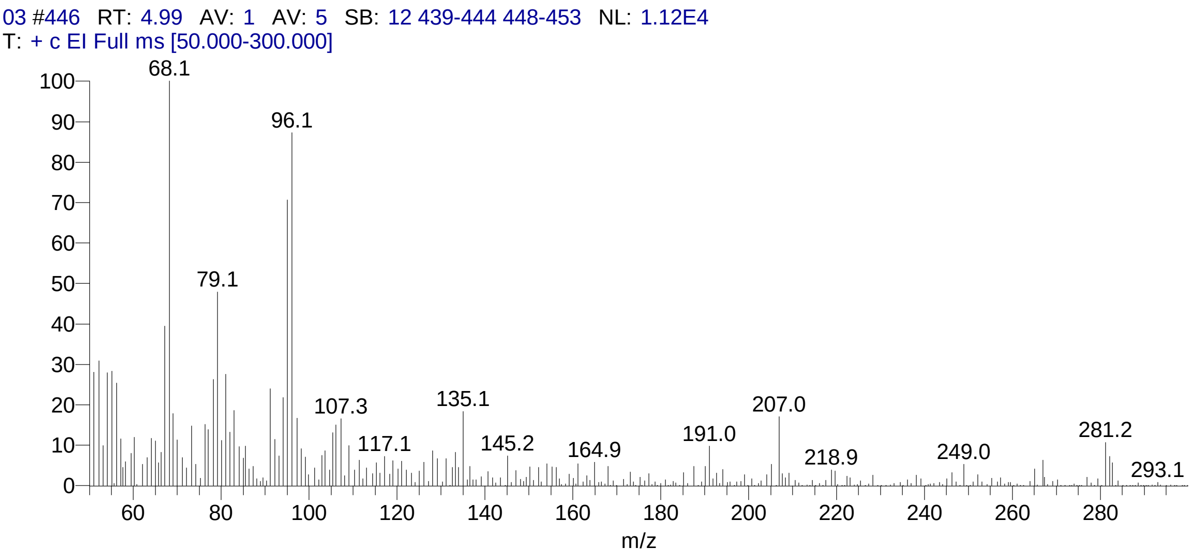

Figure 24. GC-MS spectrum of N-((3,5-dimethyl-1H-pyrazol-1-yl) methyl) thiazol-2-amine, 8

N-((3,5-dimethyl-1H-pyrazol-1-yl) methyl) pyridin-4-amine, 9

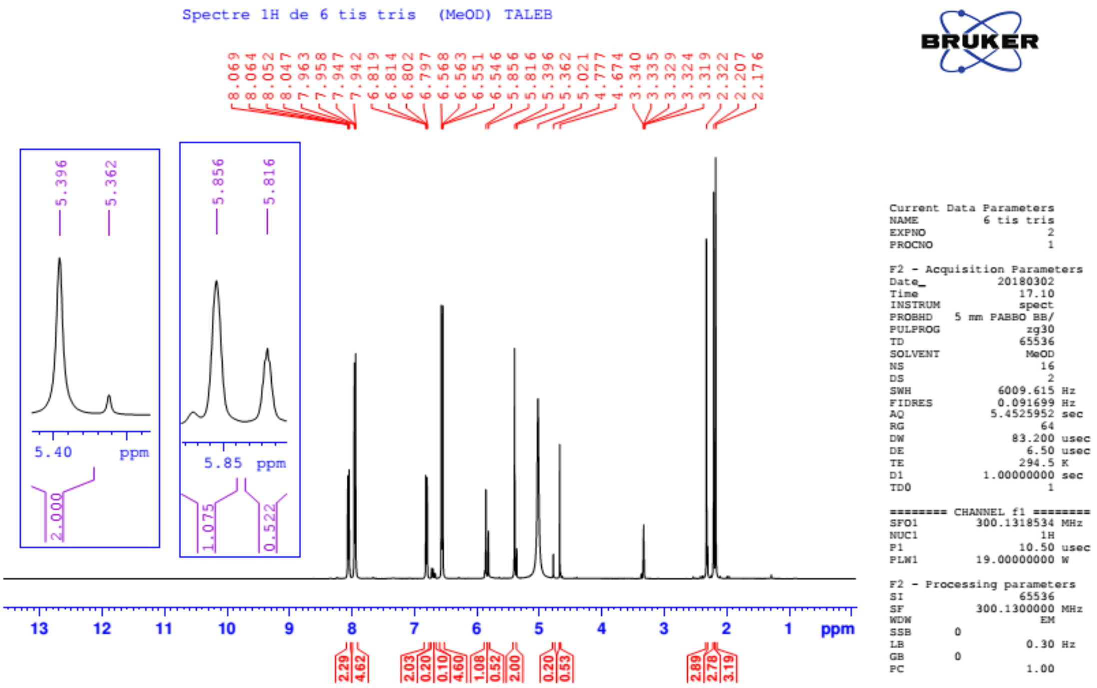

Figure 25. ^{1}H NMR spectrum of N-((3,5-dimethyl-1H-pyrazol-1-yl) methyl) pyridin-4-amine, 9

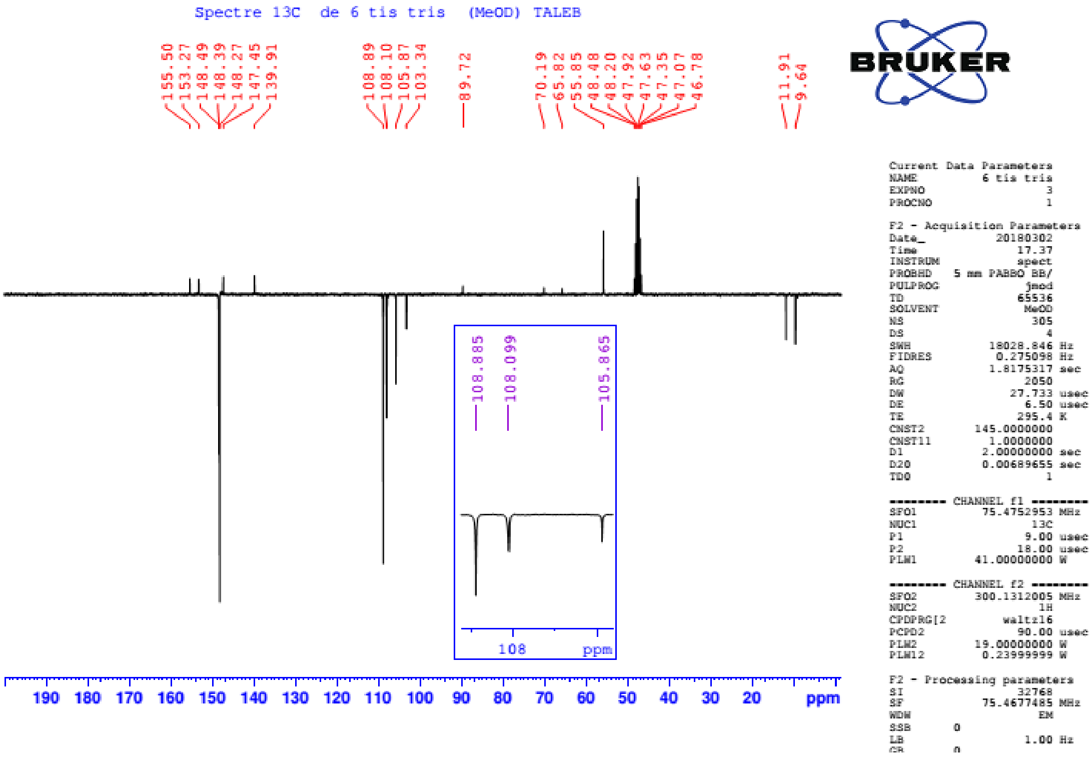

Figure 26. ^{13}C NMR spectrum of N-((3,5-dimethyl-1H-pyrazol-1-yl) methyl) pyridin-4-amine, 9

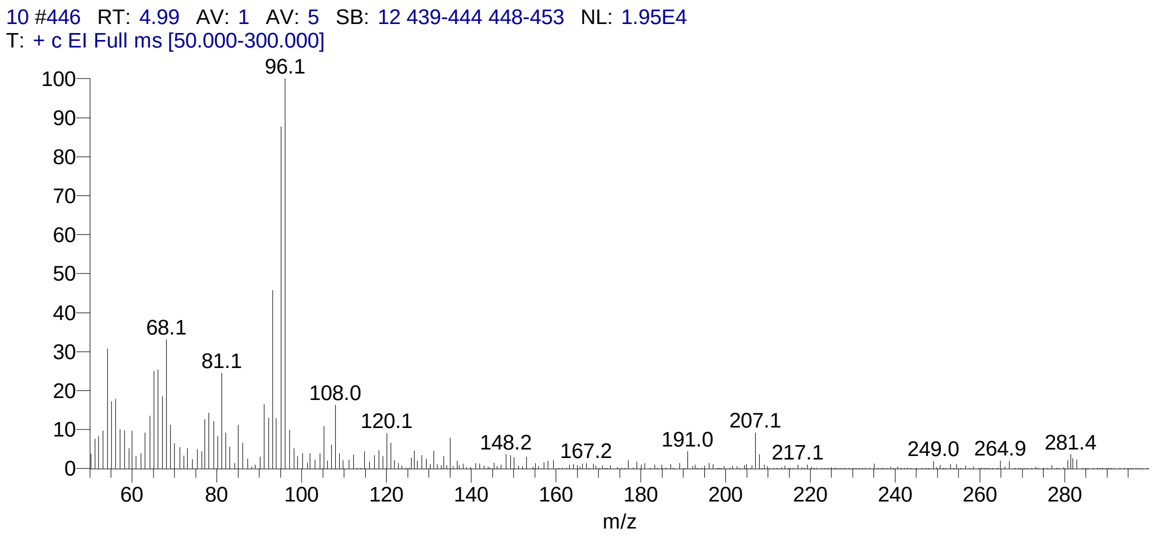

Figure 27. N-((3,5-dimethyl-1H-pyrazol-1-yl) methyl) pyridin-4-amine, 9

Ethyl 1-((1H-imidazol-1-yl) methyl)-5-methyl-1H-pyrazole-3-carboxylate, 10

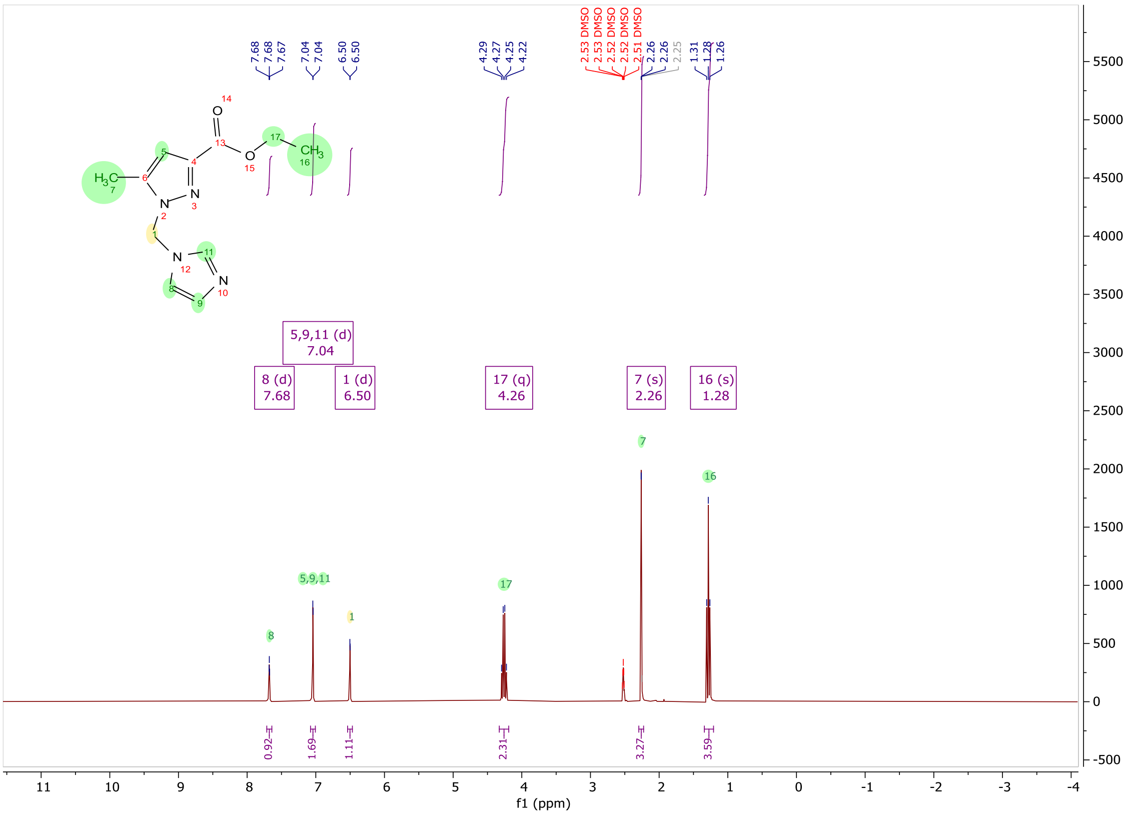

Figure 28. ^{1}H NMR spectrum of Ethyl 1-((1H-imidazol-1-yl) methyl)-5-methyl-1H-pyrazole-3-carboxylate, 10

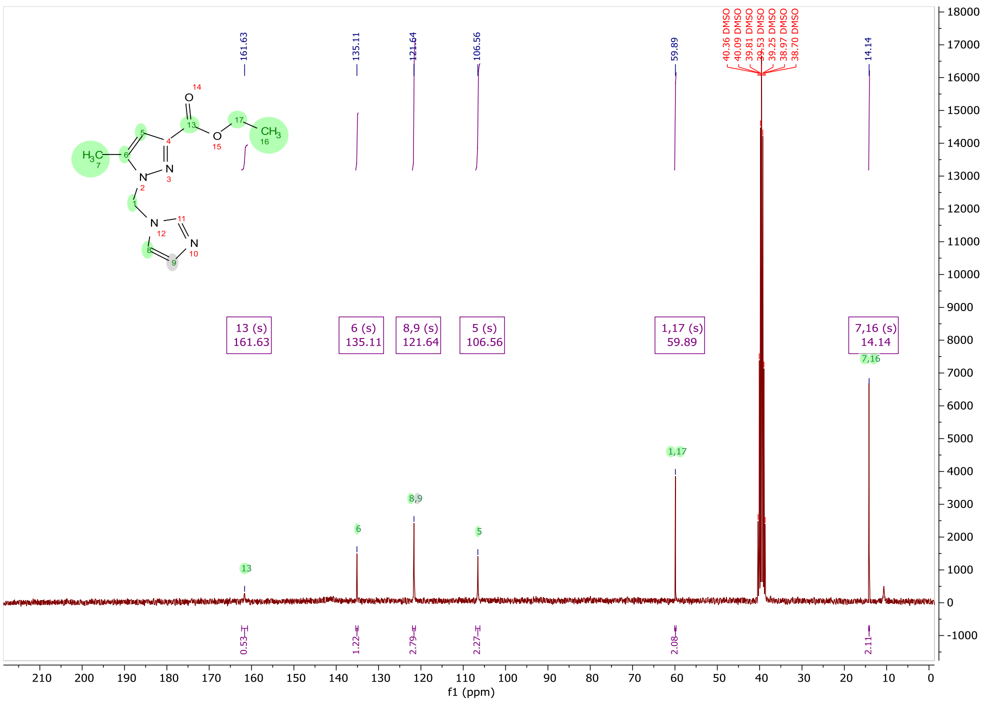

Figure 29. ^{13}C NMR spectrum of Ethyl 1-((1H-imidazol-1-yl) methyl)-5-methyl-1H-pyrazole-3-carboxylate, 10

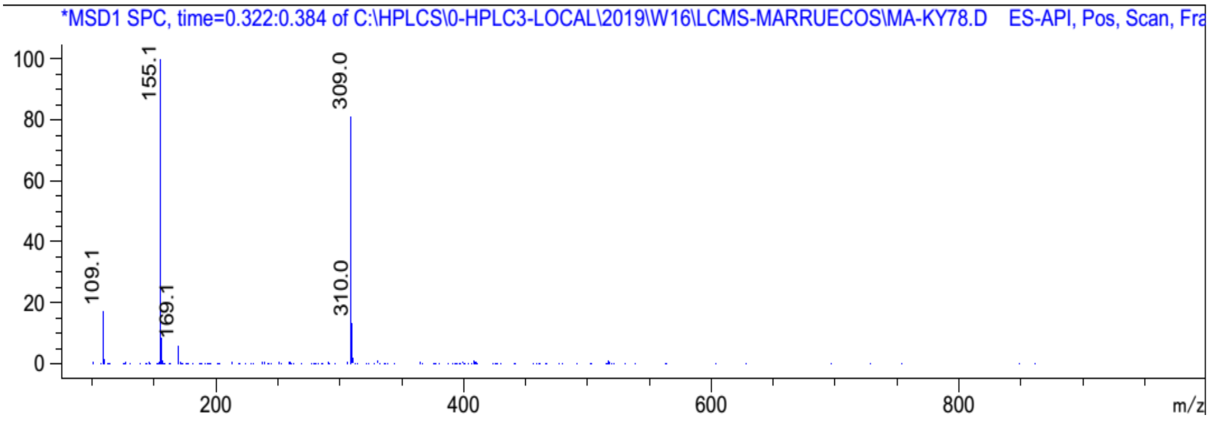

Figure 30. GC-MS spectrum of Ethyl 1-((1H-imidazol-1-yl) methyl)-5-methyl-1H-pyrazole-3-carboxylate, 10

2-(((1H-pyrazol-1-yl) methyl) amino)-6-methylpyridin-4-ol, 11

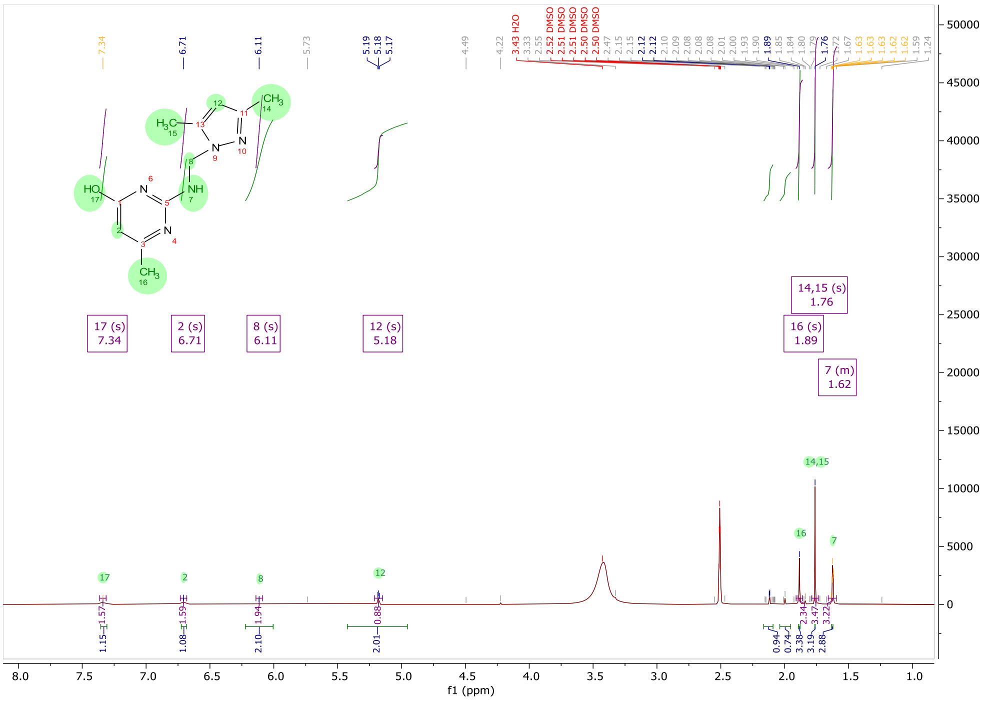

Figure 31. ^{1}H NMR spectrum of 2-(((1H-pyrazol-1-yl) methyl) amino)-6-methylpyridin-4-ol, 11

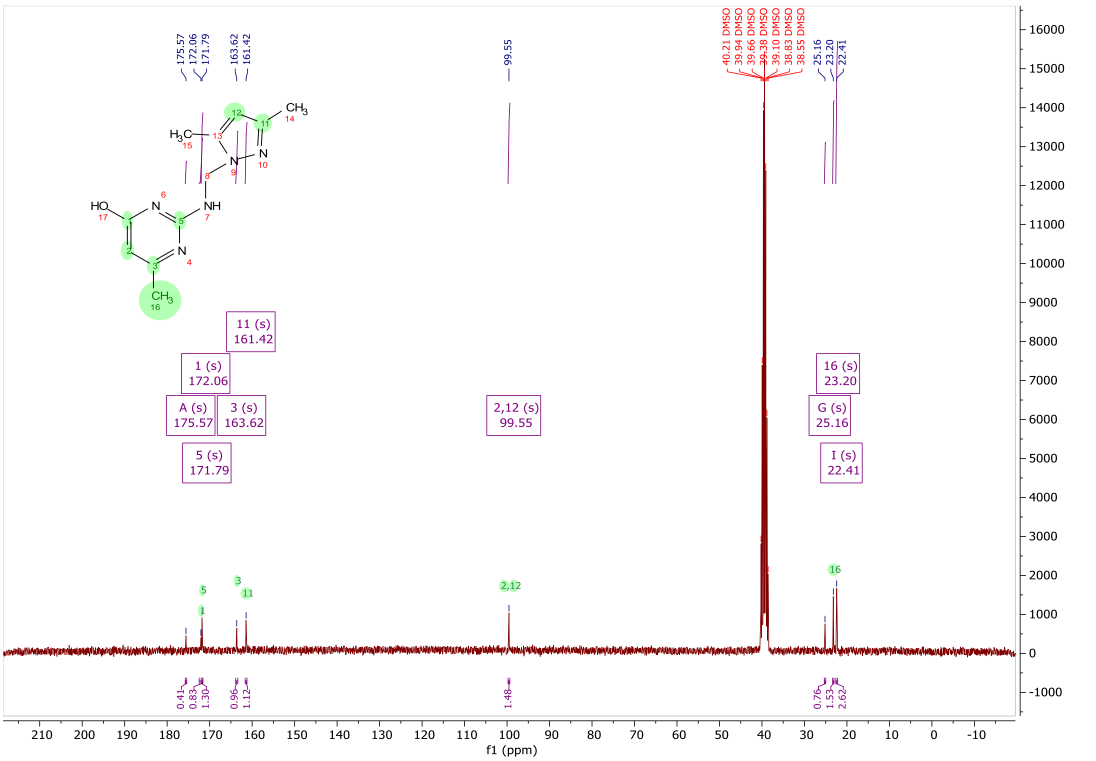

Figure 32. ^{13}C NMR spectrum of 2-(((1H-pyrazol-1-yl) methyl) amino)-6-methylpyridin-4-ol, 11

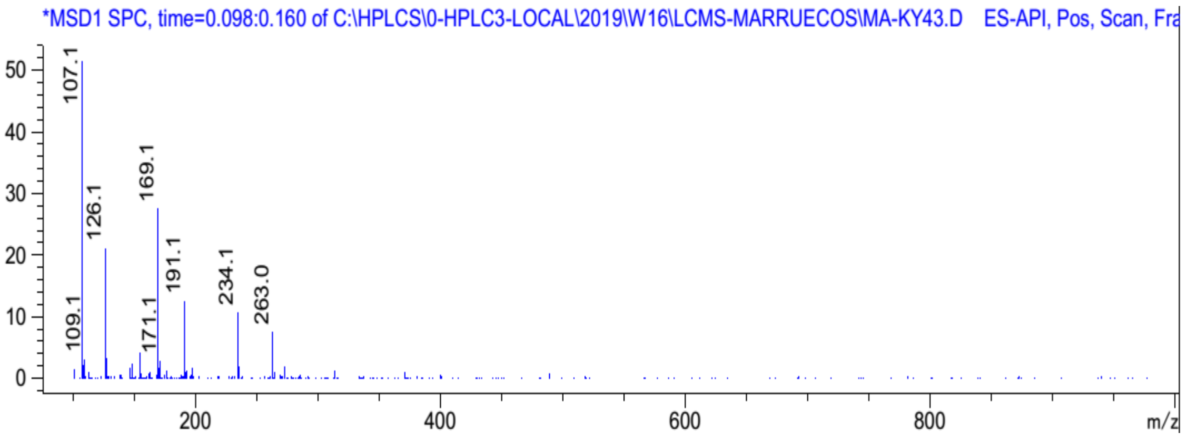

Figure 33. GC-MS spectrum of 2-(((1H-pyrazol-1-yl) methyl) amino)-6-methylpyridin-4-ol, 11

2-(((3,5-dimethyl-1H-pyrazol-1-yl) methyl) amino)-6-methylpyridin-4-ol, 12

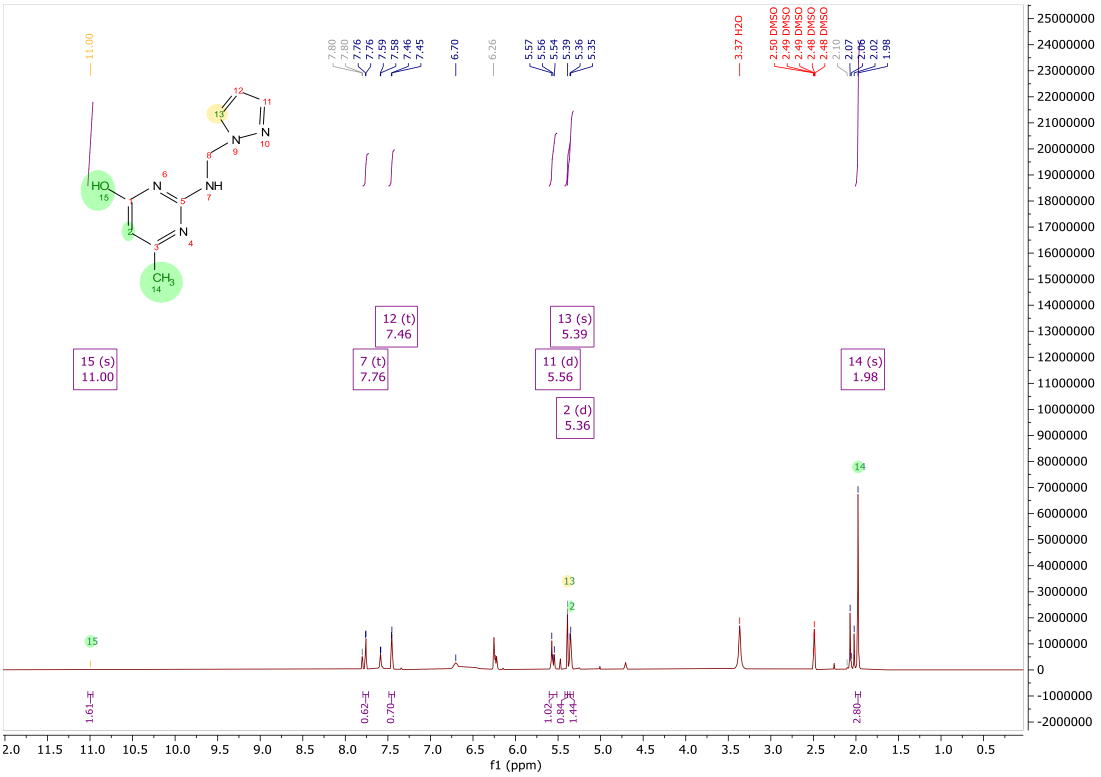

Figure 34. ^{1}H NMR spectrum of 2-(((3,5-dimethyl-1H-pyrazol-1-yl) methyl) amino)-6-methylpyridin-4-ol, 12

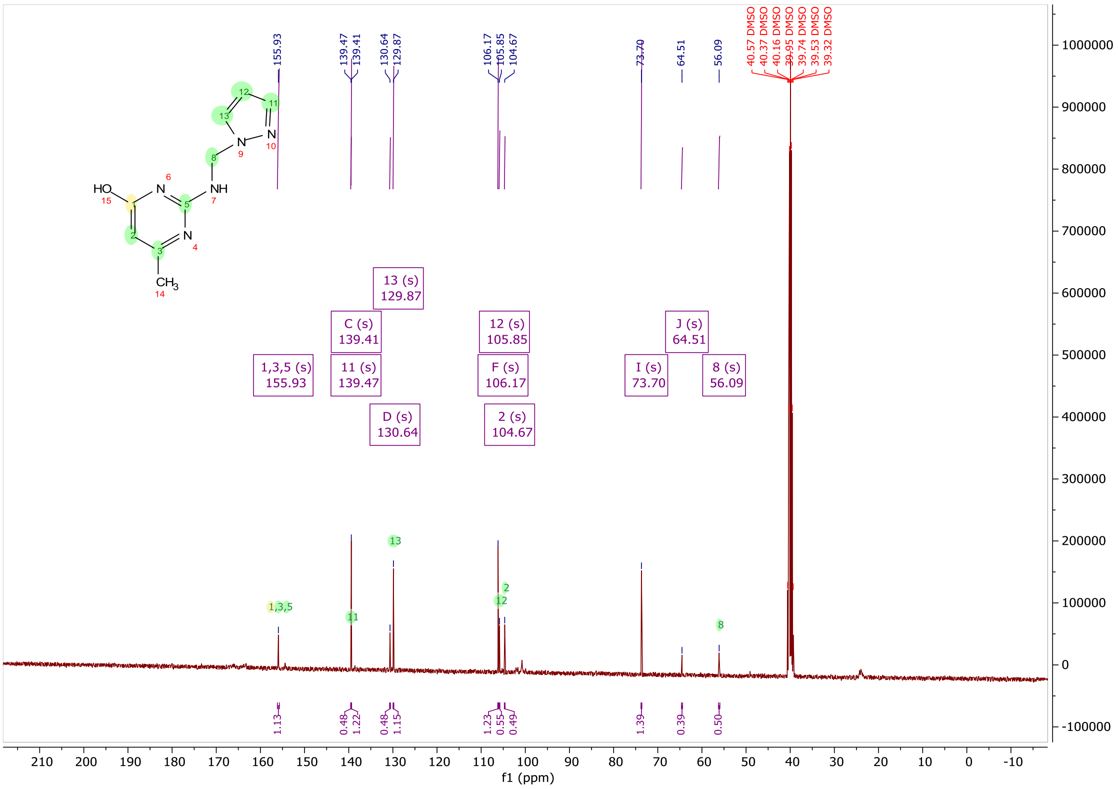

Figure 35. ^{13}C NMR spectrum of 2-(((3,5-dimethyl-1H-pyrazol-1-yl) methyl) amino)-6-methylpyridin-4-ol, 12

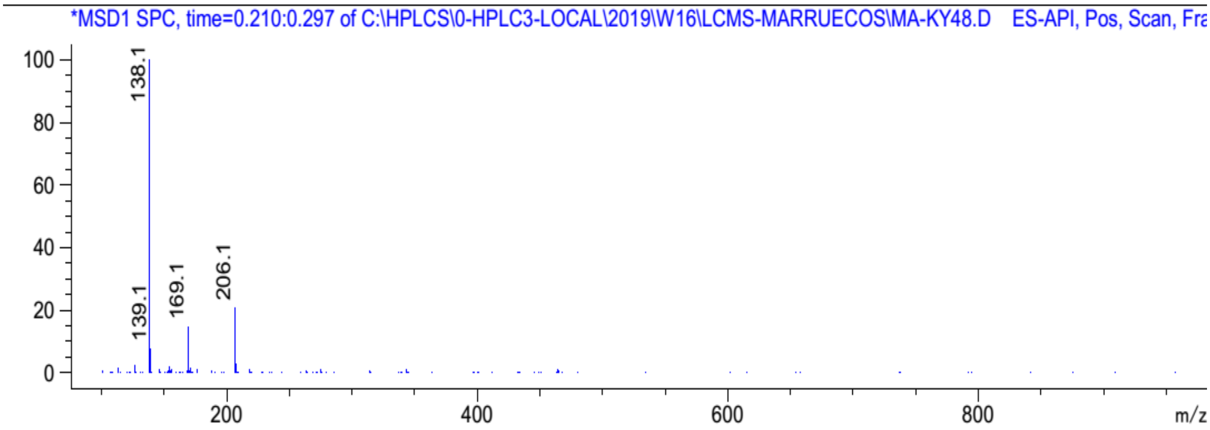

Figure 36. GC-MS spectrum of 2-(((3,5-dimethyl-1H-pyrazol-1-yl) methyl) amino)-6-methylpyridin-4-ol, 12
